# Long-term plasticity of inhibitory synapses in the hippocampus and spatial learning depends on matrix metalloproteinase 3

**DOI:** 10.1007/s00018-020-03640-6

**Published:** 2020-09-21

**Authors:** Grzegorz Wiera, Katarzyna Lebida, Anna Maria Lech, Patrycja Brzdąk, Inge Van Hove, Lies De Groef, Lieve Moons, Enrica Maria Petrini, Andrea Barberis, Jerzy W. Mozrzymas

**Affiliations:** 1 Laboratory of Neuroscience, Department of Biophysics, Wroclaw Medical University, 50-367, Wroclaw, Poland; 2 Laboratory of Cellular Neurobiology, Department of Physiology and Molecular Neurobiology, Wroclaw University, 50-205, Wroclaw, Poland; 3 Neural Circuit Development and Regeneration Research Group, Department of Biology, University of Leuven (KU Leuven), 3000 Leuven, Belgium; 4 Leuven Brain Institute (LBI), KU Leuven, Leuven, Belgium; 5 Laboratory of Synaptic Plasticity of Inhibitory Networks, Fondazione Istituto Italiano Di Tecnologia, 16163 Genoa, Italy

**Keywords:** GABA, iLTP, Mmps, Metalloproteinases, Synaptic plasticity, Synaptic inhibition

## Abstract

Learning and memory are known to depend on synaptic plasticity. Whereas the involvement of plastic changes at excitatory synapses is well established, plasticity mechanisms at inhibitory synapses only start to be discovered. Extracellular proteolysis is known to be a key factor in glutamatergic plasticity but nothing is known about its role at GABAergic synapses. We reveal that pharmacological inhibition of MMP3 activity or genetic knockout of the *Mmp3* gene abolishes induction of postsynaptic iLTP. Moreover, the application of exogenous active MMP3 mimics major iLTP manifestations: increased mIPSCs amplitude, enlargement of synaptic gephyrin clusters, and a decrease in the diffusion coefficient of synaptic GABA_A_ receptors that favors their entrapment within the synapse. Finally, we found that MMP3 deficient mice show faster spatial learning in Morris water maze and enhanced contextual fear conditioning. We conclude that MMP3 plays a key role in iLTP mechanisms and in the behaviors that presumably in part depend on GABAergic plasticity.

**Electronic supplementary material:**

The online version of this article (10.1007/s00018-020-03640-6) contains supplementary material, which is available to authorized users.

## Introduction

Learning and memory formation have been primarily associated with the plasticity of excitatory glutamatergic synapses [1], the mechanisms of which have been studied for more than four decades [2, 3]. More recently, however, inhibitory GABAergic synapses were found to exhibit many forms of long-term plasticity that is putatively important in learning and memory [4, 5]. At the circuit level, plastic changes at GABAergic synapses are thought to regulate numerous phenomena, such as the plasticity of excitatory transmission [6], timing of the auditory critical period [7], stabilization of neuronal dynamics that prevents overexcitation [8], as well as establishment, size and reciprocal interference of engrams [9, 10]. Nonetheless, a comprehensive molecular description of GABAergic plasticity has been limited by the diversity of inhibitory neurons and complexity of already known multifarious types of GABAergic plasticity that can be induced by homo- or heterosynaptic mechanisms and expressed pre- or postsynaptically.

A large body of evidence shows that the extracellular proteolysis plays a crucial role in the plasticity of glutamatergic excitatory synapses [11, 12]. For example, through the cleavage of identified proteins, matrix metalloproteinase 9 (MMP9) regulates the consolidation of long-term potentiation (LTP) [13], the structural plasticity of dendritic spines [14, 15] or synaptic plasticity that is related to addiction [16, 17] and stress-induced social impairments [18]. Moreover, another metalloproteinase—MMP3—through the cleavage of unknown substrates, has been shown to be necessary for cross-modal plasticity in the visual cortex [19], the L-type channel-dependent component of LTP [20], and the LTP of *N*-methyl-D-aspartate (NMDA) receptor transmission in the hippocampus [21]. MMP3 has been also implicated in mediating habituation-induced plasticity in the hippocampus and prefrontal cortex [22] and inhibition of this protease was suggested to alter long-term plasticity and prevent learning in the Morris water maze task [23]. However, as noted by the authors of these studies, the inhibitors that were used were not entirely specific for MMP3. Additionally, learning in the passive avoidance conditioning paradigm is associated with higher MMP3 expression in the hippocampus [24]. However, in contrast to the well-established role of extracellular proteolysis in excitatory plasticity and learning, little is known about its role in GABAergic plasticity and related cognitive functions.

The present study investigated the role of extracellular MMPs in the mechanisms of GABAergic inhibitory LTP (iLTP) in the hippocampus. We utilized a model of GABAergic iLTP, in which plasticity is induced heterosynaptically and characterized by the postsynaptic locus of expression. This form of plasticity has been described in several brain regions, including the hippocampus [25], cortex [26], and cerebellum [27]. Using electrophysiological recordings, morphological analyses of synapses, and single-particle tracking approaches, we found that the activity of MMP3 is critical for the expression of NMDA-induced iLTP and the immobilization of synaptic GABA_A_ receptors. Moreover, exogenous MMP3 activity increased amplitude of miniature inhibitory synaptic currents (mIPSCs) and the size of gephyrin synaptic clusters. Finally, we evaluated spatial learning in MMP3 deficient (*Mmp3*^−/−^) mice, which exhibited faster learning in the Morris water maze and an enhancement of contextual fear conditioning. Overall, these findings reveal that the extracellular proteolytic activity of MMP3 regulates GABAergic iLTP and shapes spatial learning.

## Results

### Induction of iLTP at CA1 inhibitory synapses depends on extracellular proteolytic activity

**Fig. 1:**
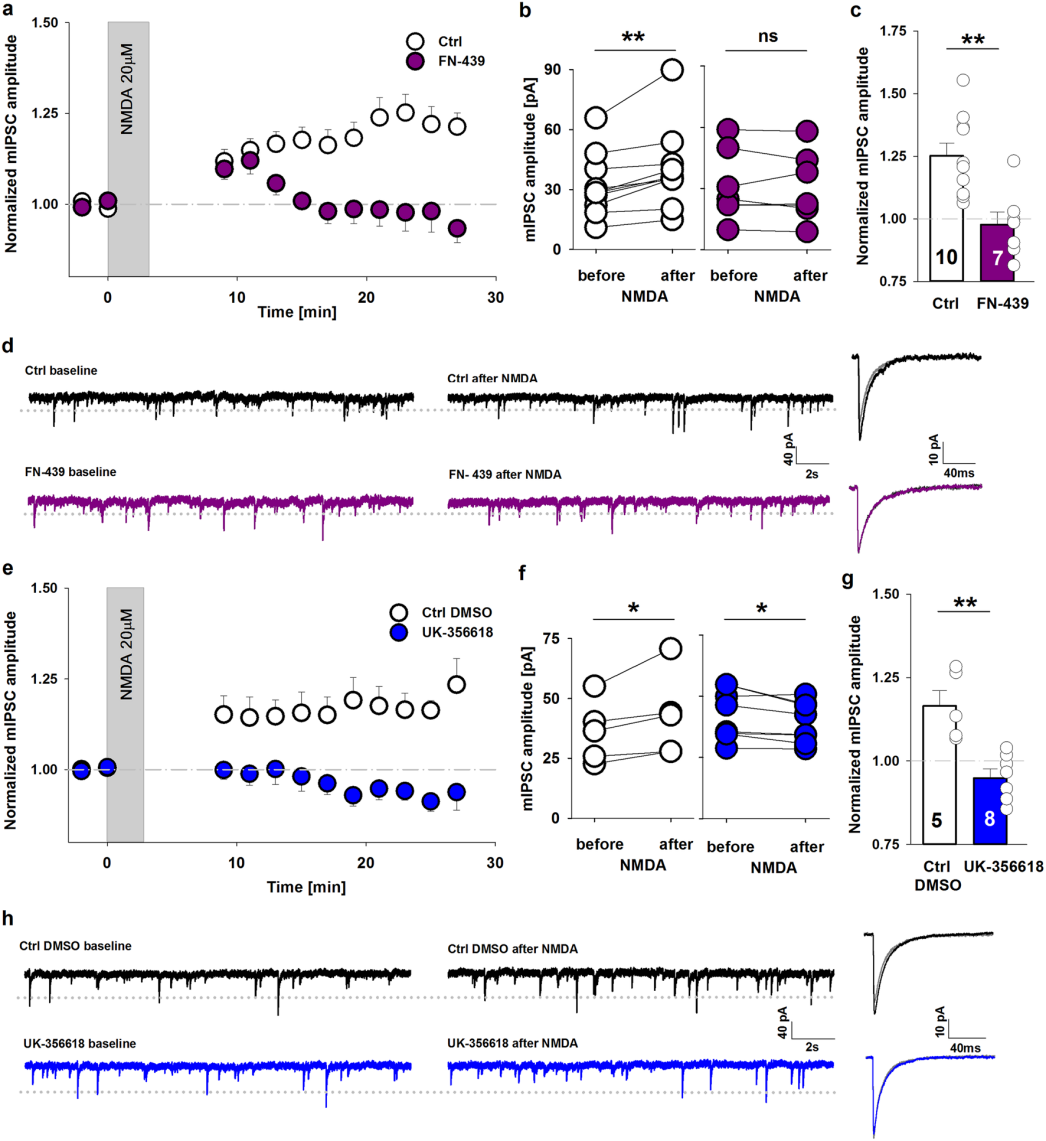
Matrix metalloproteinases are involved in GABAergic plasticity in the hippocampus. **a** Application of NMDA (3 min, 20 µM, gray bar) induced iLTP in CA1 hippocampal pyramidal cells under control conditions (white) but not in FN-439-treated slices (magenta). **b** Significant increase in mIPSC amplitude recorded from a single pyramidal cell after NMDA treatment under control conditions (paired *t* test) and the lack of this effect in the presence of FN-439. **c** Statistics for iLTP magnitude (measured 20–22 min after iLTP induction) in control (white) and FN-439-treated slices (magenta, unpaired *t* test). **d** Representative recordings and averaged traces that show the influence of NMDA application on mIPSC amplitude and frequency in control slices (top) and FN-439-treated slices (bottom). On the right side grey lines show superimposed traces before NMDA stimulation while black and magenta line represents traces after iLTP induction (in control and FN-439 treated group respectively). **e** Time course of relative mIPSC amplitude after iLTP induction, recorded from control slices (white) and in the presence of UK-356618 (blue). **f** Summary plot of mIPSC amplitudes that were recorded from control slices (white) and UK-356618-treated slices (blue) before and after NMDA application (paired *t* test). **g** Statistics of the effect of UK-356618 on the size of iLTP measured 20–22 min after NMDA stimulation (Mann–Whitney *U* test). **h** Sample traces of mIPSC recordings from an individual pyramidal cell (on the left) and averaged traces (on the right) under control conditions (top) and in the presence of UK-356618 (bottom), before and after the bath application of NMDA. In the case of averaged currents the gray lines correspond to the mIPSC recorded before the NMDA stimulation while the black and blue colors of lines represent the mIPSC measured after NMDA treatment. In b and f, the average mIPSC amplitude of each neuron is shown as one circle. The numbers on the bars refer to the number of recordings. In d and h dotted line indicates the mean amplitude of baseline mIPSC. *p < 0.05, **p < 0.01. *ns* nonsignificant

Under the present experimental conditions, GABA_A_ receptor-mediated miniature inhibitory postsynaptic current (mIPSC) amplitudes that were measured in slices from CA1 hippocampal pyramidal neurons in the whole-cell configuration were stable for at least 60 min. The mean amplitude at a holding voltage of -70 mV was 31.46 ± 3.62 pA, and the frequency was 2.18 ± 0.2 Hz. To induce postsynaptic iLTP, we adopted a chemical protocol [25] and applied NMDA (20 µM) in the bath solution for 3 min. As a result of NMDA treatment, a stable mIPSCs potentiation was observed (Fig. 1a), reaching mean amplitude enhancement of 1.25 ± 0.05 fold (relative to baseline). As explained in the “Methods” section, the extent of iLTP was assessed 20–22 min after plasticity induction and calculated relative to mIPSC amplitude values before NMDA administration (*p* = 0.003; paired *t* test; Fig. 1b). As expected [25], no changes in the relative mean frequency of mIPSCs were noticed (1.00 ± 0.08, *p* = 1; Supplementary Fig. 1a). To evaluate the involvement of extracellular proteolysis in this form of plasticity, we first used FN-439 (180 µM), a broad-spectrum MMPs inhibitor. Interestingly, in the presence of FN-439, iLTP was impaired (0.98 ± 0.05, *p* = 0.002; Fig. 1a–c). The representative raw and average traces that are shown in Fig. 1d demonstrate that NMDA stimulation increased the mIPSC amplitude under control conditions, but not when MMPs were blocked by FN-439.

In the next series of experiments, we sought to identify the type of MMP that is involved in GABAergic plasticity in our model using more specific MMPs inhibitors. Interestingly, bath application of UK-356618, which inhibits MMP3, MMP9, and MMP13, not only prevented the induction of iLTP but led also to an iLTP-to-iLTD conversion (Ctrl DMSO: 1.16 ± 0.05;UK-356618: 0.94 ± 0.03; *p* = 0.002; Fig. 1e–g). On the contrary, the MMP2/9 inhibitor SB-3CT (10 µM) had no effect on iLTP (1.18 ± 0.05; Ctrl DMSO: 1.17 ± 0.08; *p* = 0.534; Supplementary Fig. 1b, c). Altogether, this pharmacological approach provided evidence that MMP3 or MMP13 activity is required for hippocampal chemical iLTP.

### Mmp3 knockout prevents iLTP

Since the inhibitors that we used had limited selectivity for individual MMPs, the subsequent experiments evaluated iLTP in *Mmp3*^−/−^ mice [28]. We first compared the basal amplitude, frequency, rise time, and mean decay time constant of mIPSCs in slices from the *Mmp3*^−/−^ group compared with those of the wild-type (WT). Deficiency of the *Mmp3* gene did not affect mIPSC amplitude (WT: 39.07 ± 2.54 pA; *Mmp3*^*−/−*^: 42.60 ± 2.83 pA; *p* = 0.433; Supplementary Fig. 2a), frequency (WT: 2.42 ± 0.01 Hz; *Mmp3*^*−/−*^: 2.32 ± 0.098 Hz; *p* = 0.676; Supplementary Fig. 2b), or rise time (WT: 0.58 ± 0.15 ms; *Mmp3*^−/−^: 0.56 ± 0.02 ms; *p* = 0.568; Supplementary Fig. 2c), but prolonged mIPSC decay kinetics (WT: 15.54 ± 0.52 ms; *Mmp3*^−/−^: 18.14 ± 0.76 ms; *p* = 0.006; Supplementary Fig. 2d).

**Fig. 2:**
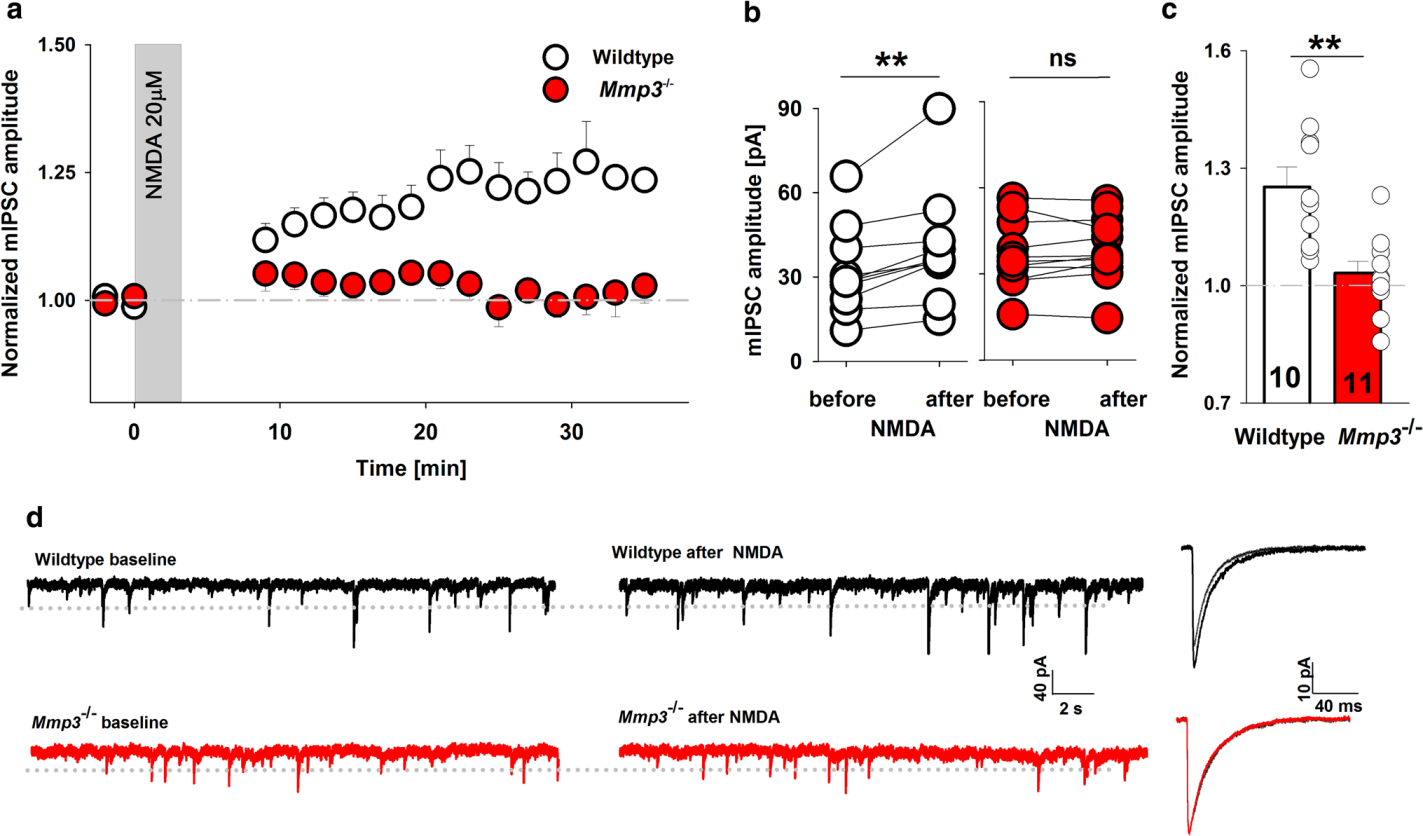
MMP3 specifically regulates iLTP induction and consolidation. **a** Time course of iLTP recorded from CA1 pyramidal cells in *Mmp3*^−/−^ mice (red) and wild-type mice (white). Note that iLTP was completely abolished in *Mmp3*^−/−^ mice. **b** Average mIPSC amplitude measured from a single pyramidal cell before and after NMDA stimulation in wild-type slices and *Mmp3*^−/−^ slices (paired *t* test). **c** Relative effect of NMDA stimulation on mIPSC amplitude in the wild-type group and *Mmp3*^−/−^ group (unpaired *t* test). **d** Examples of raw mIPSC traces recorded before and 20–22 min after iLTP induction in WT (top) and *Mmp3*^−/−^ (bottom) group and the corresponding averaged traces of their amplitude. On the right side grey lines show averaged traces before NMDA stimulation while black and red line shows traces after iLTP induction (in WT and *Mmp3*^−/−^ group respectively). On the left side the dotted line indicates the mean amplitude of baseline mIPSC. In **b**, the average mIPSC amplitude of each neuron is shown as one circle. **p < 0.01, *p < 0.05. *ns* nonsignificant. The numbers on the bars refer to the number of recordings

We next investigated the effects of MMP3 deletion on iLTP that was evoked by brief NMDA stimulation. In the *Mmp3*^−/−^ group, no iLTP was induced (relative mIPSC amplitude enhancement: 1.03 ± 0.03; *p* = 0.95; Fig. 2a–c), in contrast to the respective wild-type control (1.25 ± 0.05; *p* = 0.005; Fig. 2a–c); as also shown in Fig. 2d presenting raw and averaged exemplary traces. Interestingly, in wild-type slices, successful iLTP induction did not affect the mIPSC rise time (relative rise time: 1.05 ± 0.04; *p* = 0.21; Supplementary Fig. 3a) but prolonged the mean decay time constant (relative *τ*_mean_: 1.25 ± 0.06; *p* = 0.006; Supplementary Fig. 3b). However, in *Mmp3*^−/−^ slices, neither the mIPSC decay phase nor rise time was affected by iLTP induction (relative *τ*_mean_: 1.05 ± 0.04, *p* = 0.2; Supplementary Fig. 3a; relative rise time: 1.01 ± 0.04, *p* = 0.94; Supplementary Fig. 3b). Thus, these findings are further supporting the conclusion that the absence of MMP3 activity impaired iLTP.

### MMP3 activity is crucial for iLTP induction within a narrow time window

**Fig. 3:**
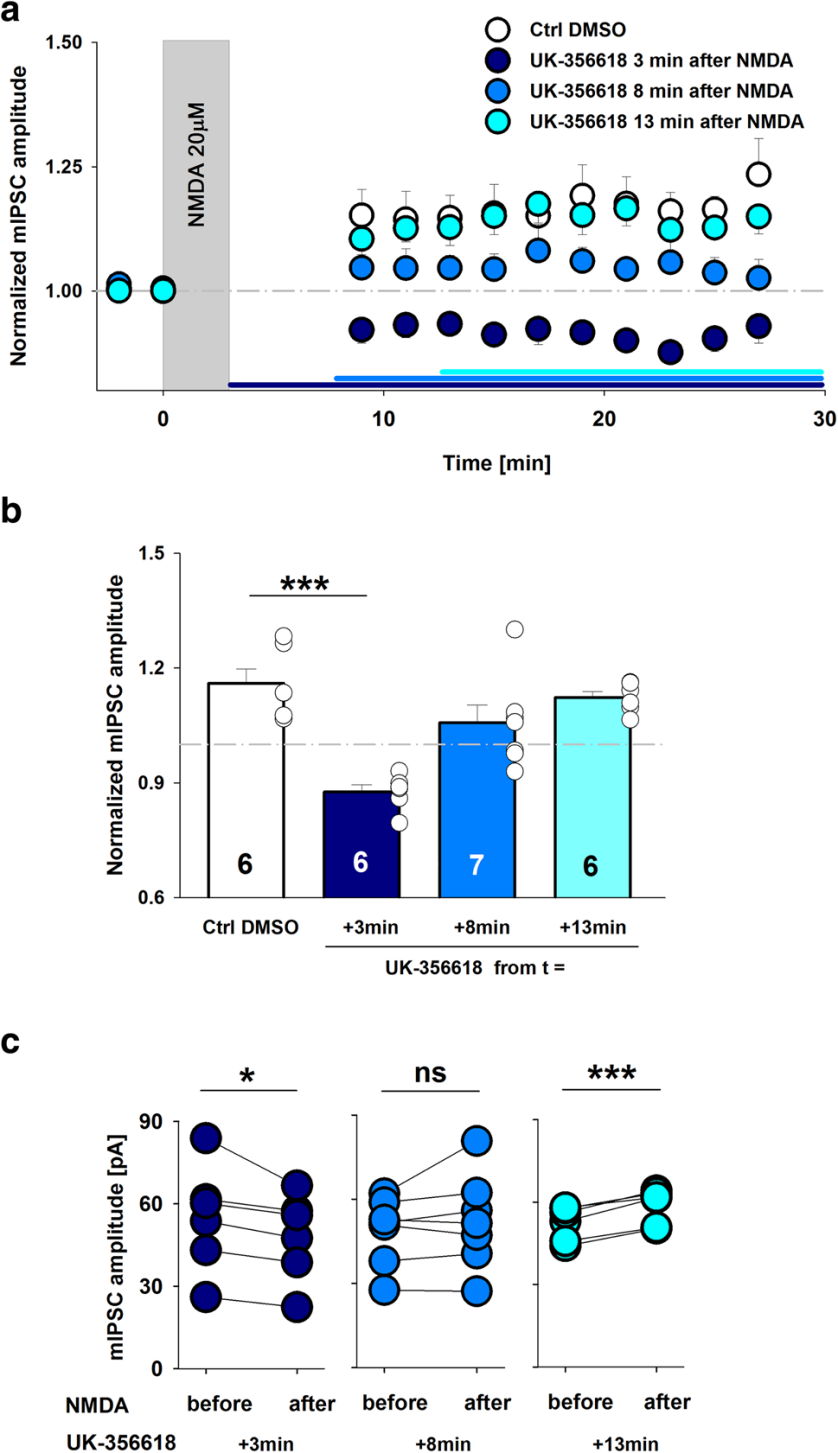
iLTP requires MMP3 activity within a restricted time window. **a** Time course of mean mIPSC amplitudes normalized to baseline values in control slices (white) and when UK-355618 was bath-applied at different time points after NMDA stimulation: 3 min (dark blue), 8 min (blue), 13 min (cyan). Note that MMP3 activity was required for iLTP up to 13 min. **b** Statistics of the ratio of mIPSC amplitudes after/before NMDA stimulation, in control slices (white) and UK-355618-treated slices at the respective time points: 3 min (dark blue), 8 min (blue), 13 min (cyan). The data were analyzed using one-way ANOVA. **c** Mean mIPSC amplitude recorded from an individual hippocampal neuron before and after NMDA stimulation in the presence of UK-356618 that was applied at varying time points relative to iLTP induction. Notice that UK-356618 that was applied 3 min after iLTP induction led to an iLTP-to-iLTD conversion (dark blue; Wilcoxon signed-rank test). UK-356618 that was applied 8 min after NMDA stimulation abolished iLTP (blue; paired *t* test). UK-356618 that was applied 13 min after NMDA stimulation had no effect on the size of iLTP (cyan; paired *t* test). ***p < 0.001, *p < 0.05. *ns* nonsignificant. The numbers on the bars refer to the number of recorded cells

Our pharmacological results demonstrated the involvement of MMP3 in the hippocampal iLTP. We next scrutinized the time window during which MMP3 activity is necessary for plasticity induction. To address this issue, we studied the impact of UK-356618 application on iLTP at varying time points after NMDA administration (3, 8, and 13 min; Fig. 3a). Application of the MMP3 activity inhibitor immediately after the end of plasticity induction led to iLTD (0.88 ± 0.02; *p* = 0.031; Wilcoxon signed-rank test in comparison to values before NMDA stimulation; Fig. 3b, c). Interestingly, this effect was similar to the previous one that we observed with UK-356618 application 15 min before NMDA stimulation (Fig. 1e). It is worth mentioning that we did not observe iLTP when we blocked MMP3 activity 8 min after NMDA stimulation began (1.06 ± 0.05; *p* = 0.264, paired *t* test; Fig. 3b, c). Interestingly, no alterations of iLTP were observed when the MMP3 inhibitor was applied 13 min after NMDA stimulation (1.12 ± 0.02; *p* < 0.001, paired *t* test; Fig. 3b, c). The data presented above show that iLTP requires MMP3 activity up to approximately 13 min after induction.

### Inhibition of MMP3 during iLTP induction impairs the recruitment of gephyrin into the synapse

**Fig. 4:**
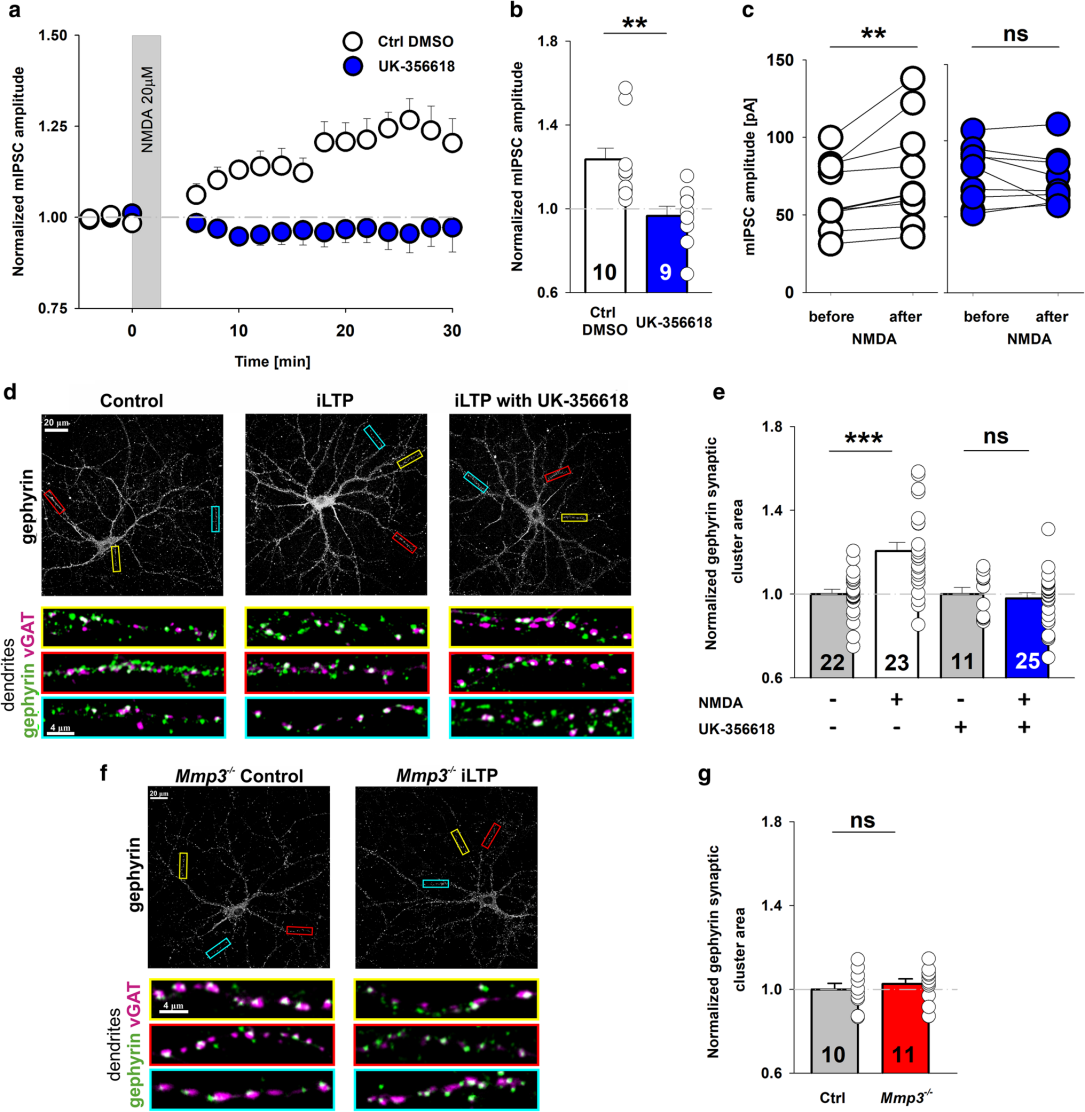
Inhibition of MMP3 activity blocks iLTP induction and gephyrin accumulation at GABAergic synapses in neuronal cultures. **a**, **b** Time course (**a**) and magnitude (**b**) of NMDA-induced iLTP recorded under control conditions and in the presence of the MMP3 inhibitor UK-356618 (*t* test) in cultured hippocampal neurons. The gray area marks the application of NMDA. **c** Changes in mIPSC amplitude measured before and 20–22 min after iLTP induction with NMDA in the control and UK-356618 groups (paired *t* test). **d**, **f** Representative confocal maximum projection images of neuronal cultures immunolabeled with antibodies against presynaptic vGAT and postsynaptic gephyrin. (Upper) Low-magnification image of the gephyrin signal in the whole neuron. Framed areas are magnified below. (Lower) Magnification of exemplary dendrites indicated above (marked with colored boxes). The colocalization of gephyrin (green) and vGAT (magenta) corresponds to the presence of gephyrin at GABAergic synapses (white). **e**, **g** Quantification of the area of synaptic gephyrin clusters in neurons that were treated with **e** sham solution, NMDA, UK-356618, and NMDA + UK-356618 (wild type) and **g** sham solution and NMDA (*Mmp3*^*−/−*^). Values are normalized to respective sham group, *t* test comparison vs. respective control. **p < 0.01, ***p < 0.001. *ns* nonsignificant. Numbers on the bars refer to the number of coverslips from at least three different batches of cultures

NMDA-induced iLTP is accompanied by an increase in accumulation of the scaffold protein gephyrin at GABAergic synapses [29]. We thus sought to investigate whether MMP3 inhibition affects synaptic clustering of gephyrin using an immunocytochemical approach. We used cultures of primary hippocampal neurons, which allowed us to analyze distinct discernible clusters of gephyrin in individual dendrites and synapses. To determine whether our major findings could be reproduced in this model, we evaluated iLTP induction and its sensitivity to MMP blockers in cultured neurons. Similar as in slice recording, the MMP2 and MMP9 inhibitor SB-3CT did not affect GABAergic plasticity (Supplementary Fig. 4a–c) while in the presence of UK-356618, cultured neurons did not undergo iLTP (ratio of mIPSC amplitude after/before iLTP; control with DMSO: 1.21 ± 0.05; UK-356618: 1.00 ± 0.03; *p* = 0.008; Fig. 4a–c). We next evaluated NMDA-induced gephyrin modifications by immunolabeling gephyrin and vesicular GABA transporter (vGAT) to reveal GABAergic synapses (Fig. 4d, f). The size and fluorescence intensity of synaptic gephyrin clusters were analyzed under control conditions and 20 min after iLTP induction, both in wild-type and *Mmp3*^*−/−*^ mice. As previously reported [30], the bath application of NMDA increased the average size of synaptic gephyrin clusters by ~ 20% (area normalized to control; sham: 1.00 ± 0.02; iLTP: 1.21 ± 0.04; *p* < 0.001; Fig. 4e), whereas NMDA stimulation in the presence of UK-356618 did not enlarge synaptic gephyrin clusters (sham with UK-356618: 1.00 ± 0.03; iLTP with UK-356618: 0.98 ± 0.03; *p* = 0.64; Fig. 4e). Furthermore, we observed that in *Mmp3*^*−/−*^ neurons, the application of NMDA did not cause changes in the size of gephyrin clusters, similar to the UK-356618-treated group (*Mmp3*^*−/−*^ sham: 1.00 ± 0.03; *Mmp3*^*−/−*^ iLTP: 1.03 ± 0.02; *p* = 0.50; Fig. 4g). In parallel, iLTP induction left the average fluorescence intensity of synaptic gephyrin clusters unchanged (Supplementary Fig. 4d, e). These results indicate that functional iLTP is accompanied by structural changes at GABAergic synapses, and both functional and structural manifestations of iLTP can be blocked by inhibiting the proteolytic activity of MMP3.

### Immobilization of GABAA receptors at inhibitory synapses depends on MMP3 activity

A key mechanism that regulates the number of GABA_A_ receptors at inhibitory synapses relies on the ability of synapses to trap receptors that laterally diffuse between synaptic and extrasynaptic domains [31]. At GABAergic synapses, iLTP induction is accompanied by a reduction and more confined diffusion of synaptic GABA_A_ receptors [30]. Therefore, we tested whether the proteolytic activity of MMP3 plays a role in GABA_A_ receptor immobilization, which could underlie iLTP in our model. The diffusion of GABA_A_ receptors was studied in cultured hippocampal neurons by imaging quantum dots (QDs) that were tethered to the α1 subunit of GABA_A_ receptors. Inhibitory synaptic localization was identified by live immunostaining of the presynaptic marker vGAT.

**Fig. 5:**
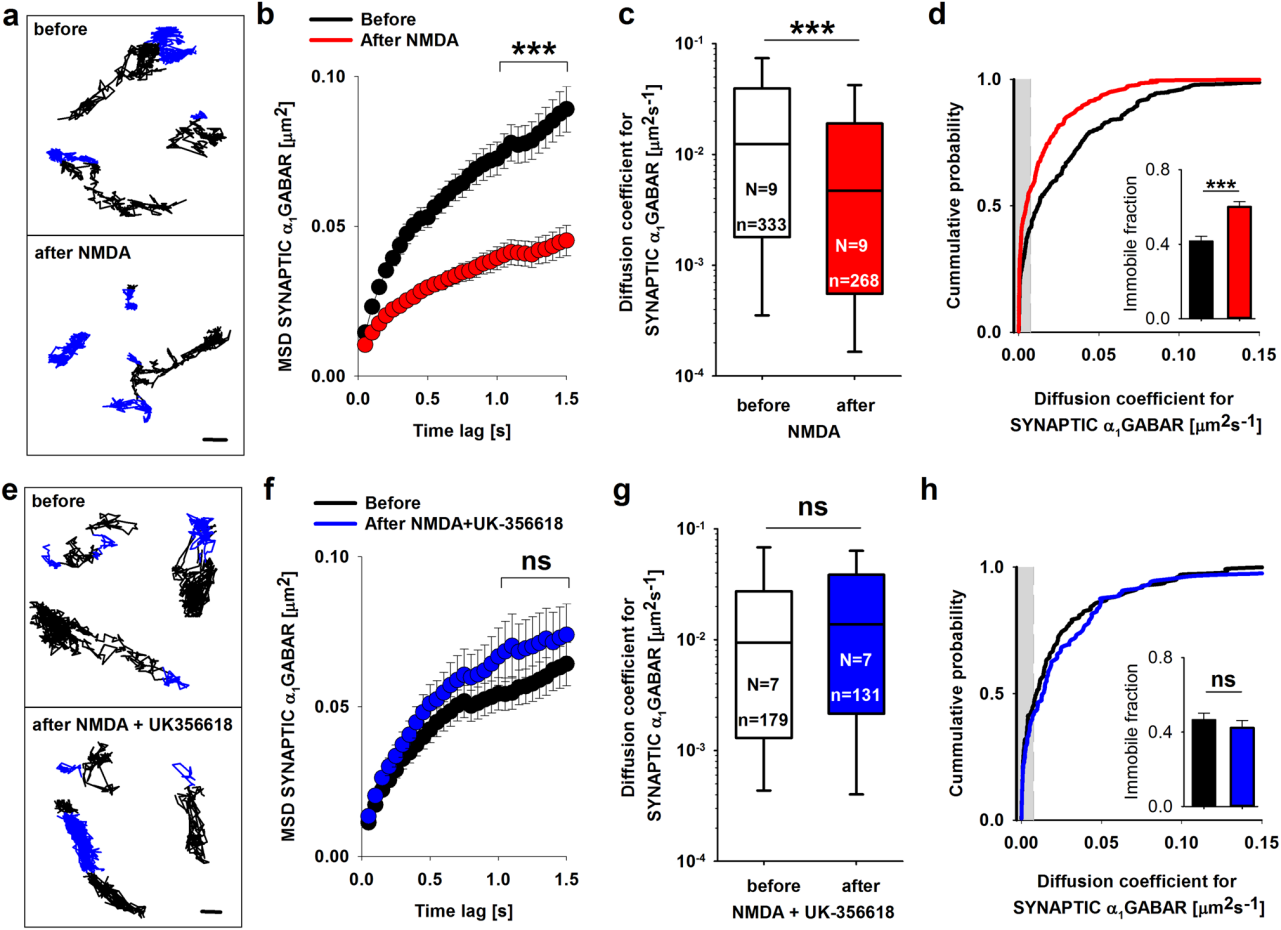
Immobilization of α_1_GABA_A_ receptors at GABAergic synapses critically depends on MMP3 activity. **a** Representative trajectories of individual α_1_-containing GABA_A_ receptors diffusing in extrasynaptic membrane (black) and in synaptic region (blue) identified by live vGAT staining. Scale bar 500 nm. **b** Mean square displacement of synaptic α_1_GABA_A_ receptors before (black) and 20 min after (red) NMDA stimulation. **c** Interquartile range (IQR; 25–75%) and median diffusion coefficient of synaptic α_1_GABA_A_ receptors before and 20 min after NMDA stimulation. **d** Cumulative probability distributions of diffusion coefficients for synaptic α_1_GABA_A_ receptors before (black) and 20 min after (red) NMDA stimulation. The gray area marks the part of the cumulative distribution that contains immobile receptors (*D* < 0.0075 µm^2^s^−1^). (Inset) Comparison of the immobile fraction before and after iLTP. **e** Representative trajectories of individual α_1_-containing GABA_A_ receptors diffusing in extrasynaptic membrane (black) and in synaptic region (blue) acquired before and 20 min after MMP3 infusion. Scale bar 500 nm. **f** Mean square displacement of synaptic α_1_GABA_A_ receptors before (black) and 20 min after (blue) NMDA stimulation with UK-356618. **g** Interquartile range and median diffusion coefficient of synaptic α_1_GABA_A_ receptors before and 20 min after NMDA stimulation in the presence of UK356618. **h** Cumulative probability distributions of diffusion coefficients for synaptic α_1_GABA_A_ receptors before (black) and 20 min after (blue) NMDA stimulation in the presence of UK-356618. The gray area marks the part of the cumulative distribution that contains immobile receptors (*D* < 0.0075 µm^2^s^−1^). (Inset) Comparison of the immobile fraction before and after iLTP induced in the presence of UK-356618. ***p < 0.001. *ns* nonsignificant. The data in **b**, **c**, **d**—inset, **f**, **g**, and **h**—inset, were analyzed using the Mann–Whitney *U* test. *N* and *n* refer to the number of coverslips and analyzed trajectories, respectively

We compared the diffusion properties of synaptic GABA_A_ receptors before and 20 min after iLTP induction with NMDA. This analysis revealed that sham treatment (i.e., only vehicle treatment) did not affect the diffusion of α_1_GABA_A_ receptors, the fraction of immobile receptors, or the mean squared displacement (MSD; Supplementary Fig. 5a–c). In contrast, comparisons of MSD plots before and 20 min after NMDA stimulation indicated that iLTP induction resulted in a more confined diffusion of synaptic α_1_GABA_A_ receptors (Fig. 5a, b) [29]. iLTP induction with NMDA significantly slowed synaptic GABA_A_ receptors, reflected by a lower diffusion coefficient (median and interquartile range [IQR] before: 0.0124, 0.0018–0.0393 μm^2^s^−1^; median and IQR after: 0.0047, 0.0006–0.0190 μm^2^s^−1^; *p* < 0.001; Fig. 5c) and a prolonged duration of immobile periods (immobile fraction) (before: 0.41 ± 0.03; after: 0.61 ± 0.03; *p* < 0.001; Fig. 5d). To further test the effect of MMP3 inhibition on GABA_A_ receptor diffusion, we induced iLTP in the presence of UK-356618. The inhibition of MMP3 activity prevented the increase of confinement of synaptic trajectories (Fig. 5e, f), prevented the reduction of the diffusion coefficient (median and IQR before: 0.0094, 0.0013–0.0270 μm^2^s^−1^; median and IQR after: 0.0138, 0.0022–0.0384 μm^2^s^−1^; *p* = 0.24; Fig. 5g) and prevented the increase of immobilization of GABA_A_ receptors (immobile fraction before: 0.48 ± 0.03; after: 0.42 ± 0.02; *p* = 0.41; Fig. 5h), and, thus leaving receptor lateral diffusion unaffected by the iLTP induction protocol. Treatment with UK-356618 alone (without NMDA) did not affect the diffusion properties of synaptic α_1_GABA_A_ receptors (Supplementary Fig. 5d–f). These results suggest that the lateral diffusion of GABA_A_ receptors during the iLTP is modulated by MMP3-mediated extracellular proteolytic activity.

### Exogenous active MMP3 induces plasticity at GABAergic synapses

**Fig. 6:**
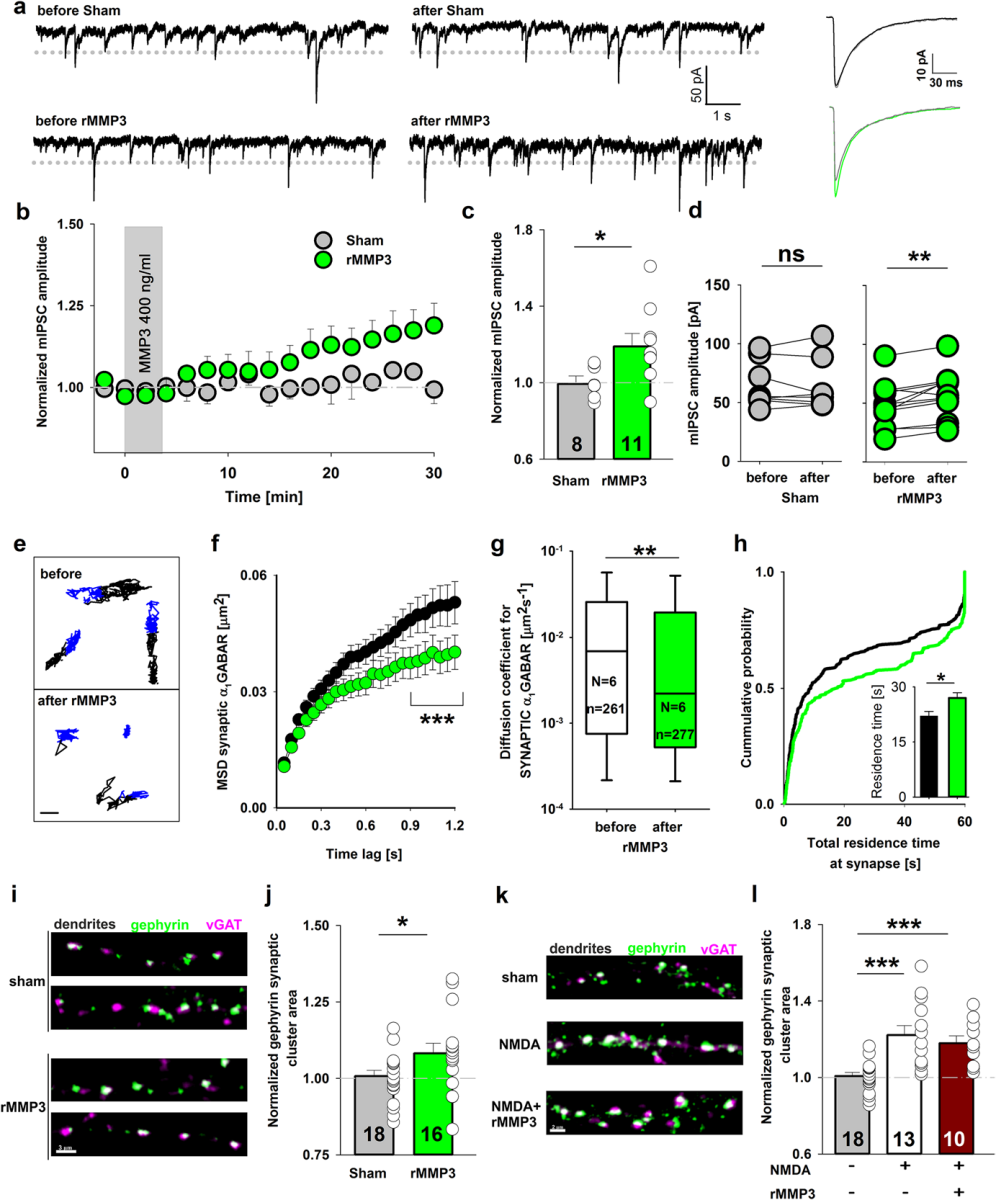
Short-term application of active rMMP3 potentiates mIPSC amplitude, immobilizes synaptic GABA receptors and increases the size of synaptic gephyrin clusters. **a** On left: typical raw mIPSC recordings before and 30 min after rMMP3 or sham treatment. Dotted line represents the man amplitude of baseline mIPSC. On right: averaged mIPSC traces recorded before (grey) and 30 min after treatment (sham—black, rMMP3—green). **b** mIPSC amplitude recorded in the sham-treated group and in neurons that were incubated for 3 min with active rMMP3 (400 ng/ml). **c** Comparison of changes in mIPSC amplitude in the sham group and in neurons that were treated with rMMP3. **d** Changes in mIPSC amplitude measured before and 28–30 min after rMMP3 administration (green) or sham treatment (gray). **e** α_1_GABA_A_ receptor trajectories before and after application of rMMP3. Extrasynaptic parts of trajectories are marked as black, synaptic parts are marked blue. Scale bar 500 nm. **f** Mean square displacement of synaptic α_1_GABA_A_ receptors before (black) and 20 min after (green) rMMP3 incubation. **g** Interquartile range and median diffusion coefficient of synaptic α_1_GABA_A_ receptors before and 20 min after short-term (2 min) incubation with rMMP3. *N* and *n* refer to the number of coverslips and analysed trajectories, respectively. **h** Cumulative probability distributions of the total residence time in the synapse by α_1_GABA_A_ receptors before (black) and 20 min after (green) the application of rMMP3. The total imaging time (1 min) indicates the maximal possible time that the analysed receptors could be detected in the synapse. (Inset) Average total residence time before and after rMMP3 treatment. **i** Representative confocal maximum projection images obtained from neuronal cultures that were immunolabeled with antibodies against presynaptic vGAT (magenta) and postsynaptic gephyrin (green). The colocalization of gephyrin and vGAT (white) corresponds to the presence of gephyrin at GABAergic synapses. (Upper) Images of two typical portions of dendrites after sham treatment. (Lower) Images of two typical portions of dendrites that undergo 2 min 15 s incubation with rMMP3 (400 ng/ml). **j** The average area of gephyrin clusters at dendritic inhibitory synapses (normalized to sham). **k** Representative confocal maximum projection images obtained from neuronal cultures that were labeled with antibodies against presynaptic vGAT (magenta) and postsynaptic gephyrin (green). The upper, middle, and lower images show typical fragments of dendrites that were fixed 20 min after sham, NMDA, and NMDA + rMMP3 treatment, respectively. **l** Quantification of the average area of synaptic gephyrin clusters in neuronal cultures that were treated with sham solution, NMDA, and NMDA + rMMP3. Values are normalized to sham group. *p < 0.05, **p < 0.01, ***p < 0.001. *ns* nonsignificant. The data in **c**, **j**, **l**, were analyzed using *t* test vs. respective control. The data in **d** were analyzed using paired *t* tests. The data in **f–h** were analyzed using the Mann–Whitney *U* test. Numbers on the bars refer to the number of coverslips from at least three different batches of cultures

Considering that inhibition of endogenous MMP3 blocked the expression of postsynaptic GABAergic iLTP, we next investigated whether exogenous active recombinant rMMP3 affects inhibitory synaptic transmission. Brief treatment for 3 min (see “Methods” sections for a description of used treatment durations) with active rMMP3 (400 ng/ml) in neuronal cultures progressively increased the mIPSC amplitude, which reached on average 119% of baseline after 28–30 min (Fig. 6a–c). Comparisons of mIPSC amplitudes before and 28–30 min after incubation with rMMP3 indicated significant potentiation (before: 47.6 ± 5 pA; after: 56.0 ± 5 pA; *p* = 0.002; Fig. 6d) that was maintained throughout the rest of the recording period (up to 60 min in the most stable recordings; Supplemental Fig. 6a). In the sham group (i.e., treated only with vehicle), we did not observe changes in mIPSC amplitude (before: 64.7 ± 7 pA; after: 63.9 ± 8 pA; *p* = 0.74; Fig. 6d). Neither sham treatment nor rMMP3 application changed the mIPSC frequency (Supplemental Fig. 6b). Altogether, these results indicate that MMP3 activity induces the potentiation of inhibitory synaptic transmission that lasts for at least 1 h. Such potentiation may be regarded as chemically induced LTP and referred to as MMP3-iLTP.

We next investigated whether the diffusion of synaptic GABA_A_ receptors is directly modulated by exogenous MMP3 activity. We performed single-particle tracking of α_1_GABA_A_ receptors in neurons that were acutely treated for 2 min with active rMMP3 (Fig. 6e; see “Methods” section for a description of used treatment durations). The MSD *vs*. time plot of synaptic trajectories reached a lower plateau after MMP3 treatment, suggesting more confined α_1_GABA_A_ receptor diffusion (Fig. 6f). Furthermore, the median of the diffusion coefficient of synaptic receptors changed from 0.0069 μm^2^s^−1^ before to 0.0022 μm^2^s^−1^ 20 min after MMP3 treatment (IQR before: 0.00075–0.0252 μm^2^s^−1^, after: 0.00052–0.0194 μm^2^s^−1^; *p* = 0.008; Fig. 6g). After rMMP3 application, the distribution of diffusion coefficients shifted to significantly lower values, with a higher immobile fraction (before: 0.53 ± 0.03, after: 0.61 ± 0.03 *p* = 0.018; Supplemental Fig. 7b) and a longer residence time at the synapse (before: 22 ± 1 s; after: 27 ± 1 s; *p* = 0.011; Fig. 6h). This result was corroborated by matched observations of the same GABA_A_ receptors identified at the synapse before and 20 min after rMMP3 infusion, indicating a significant decrease in the diffusion coefficient (Supplemental Fig. 7a). The diffusion parameters of extrasynaptic α_1_GABA_A_ receptors were comparable before and after MMP3 treatment (Supplemental Fig. 7c, d). These results indicate that the proteolytic activity of MMP3 promotes the synaptic trapping of GABA_A_ receptors, resulting in potentiation of the GABAergic synapse.

### Potentiation induced by active rMMP3 occludes NMDA-induced iLTP

To further investigate the effect of exogenous rMMP3 activity on inhibitory synapses, we analyzed the size and average fluorescence intensity of synaptic gephyrin clusters (Fig. 6i). Active rMMP3 application for 2 min 15 s increased the mean size of synaptic (i.e., vGAT-positive) gephyrin clusters compared with sham-treated neurons (area normalized to control; sham: 1.00 ± 0.02; MMP3-iLTP: 1.08 ± 0.03; *p* = 0.029; Fig. 6j). rMMP3 treatment decreased also the average fluorescence intensity of gephyrin synaptic clusters (Supplemental Fig. 7e). Overall, the effect of exogenous active rMMP3 on the area of gephyrin clusters further substantiates the notion that extracellular proteolysis might regulate the strength of GABAergic synapses.

In the next series of experiments, we investigated the relationship between NMDA-induced iLTP and MMP3-iLTP to determine whether MMP3-iLTP occludes NMDA-induced iLTP. We reasoned that if MMP3-iLTP and NMDA-induced iLTP arise from two separate molecular mechanisms, then we should observe an additive effect on gephyrin cluster area when both NMDA and MMP3 are applied together. However, the application of NMDA together with exogenous rMMP3 increased the size of synaptic gephyrin clusters to a similar level as it was observed after treatment with NMDA alone (area normalized to control; sham: 1.00 ± 0.02; NMDA: 1.22 ± 0.05; NMDA + rMMP3: 1.18 ± 0.04; NMDA *vs*. NMDA + rMMP3 *p* = 0.528; Fig. 6k, i). Neither NMDA alone nor NMDA + rMMP3 altered the average fluorescence intensity of gephyrin synaptic clusters (Supplemental Fig. 7f). These results suggest that both the activity of NMDA receptors and MMP3-dependent proteolysis participate in the same molecular mechanism that is responsible for iLTP induction.

### Exogenous rMMP3 rescues impairment in iLTP in MMP3-deficient neurons

**Fig. 7:**
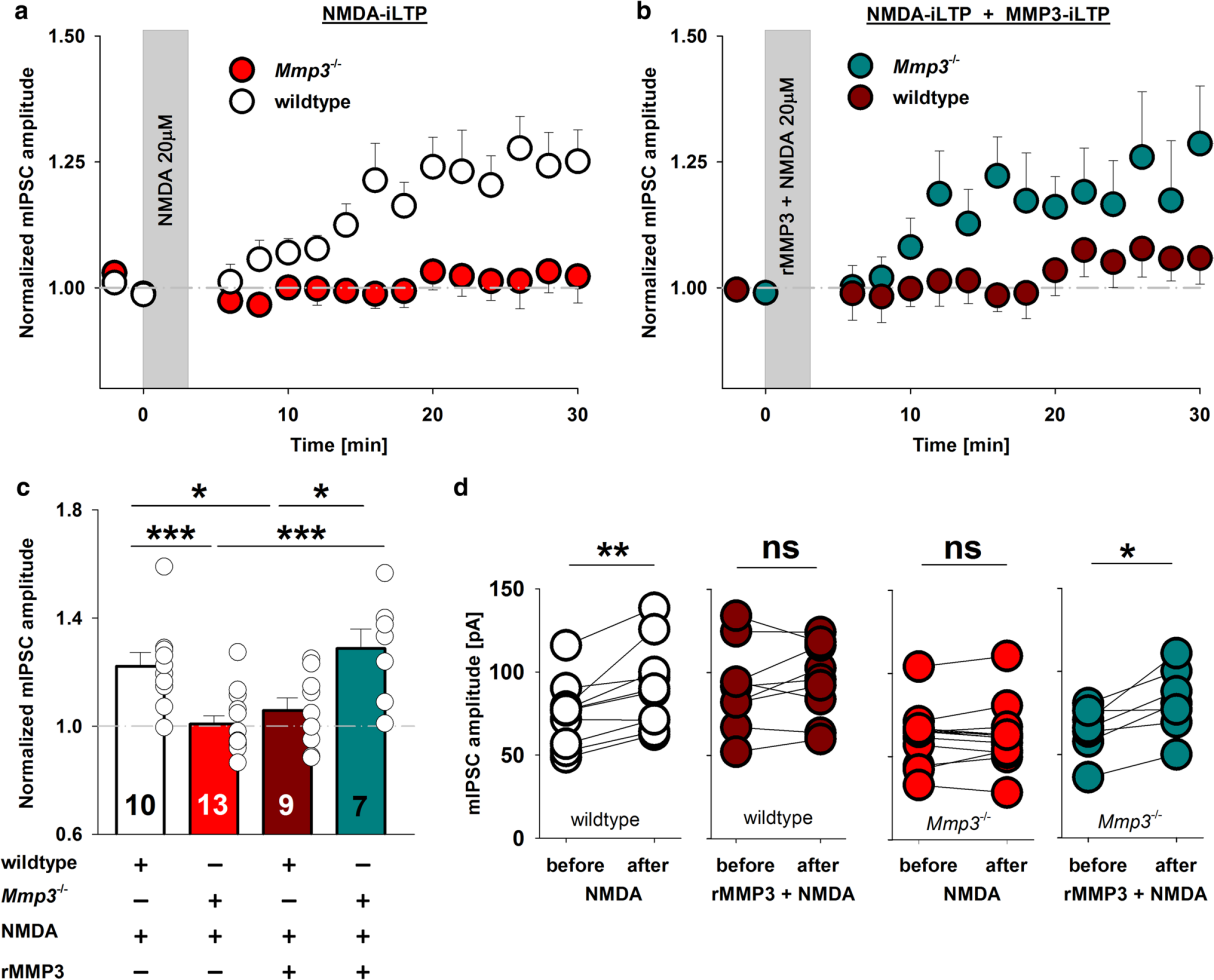
Short-term application of exogenous active rMMP3 together with NMDA restores iLTP in *Mmp3*^−/−^ neurons and impairs iLTP in wild-type neuronal cultures. **a** Time course of NMDA-induced iLTP recorded in hippocampal neuronal cultures that were prepared from wild-type (white) and *Mmp3*^−/−^ (red) mice. The gray area marks the application of NMDA. **b** Time course of iLTP that was induced with NMDA coapplied with active rMMP3 (400 ng/ml) for 2 min 15 s in hippocampal neuronal cultures from wild-type (brown) and *Mmp3*^−/−^ (dark cyan) mice. The gray area marks the application of NMDA with MMP3. **c** Comparison of changes in mIPSC amplitude after iLTP induction in the respective groups (colors as in **a** and **b**). **d** mIPSC amplitude measured before and 20–22 min after iLTP induction (colors as in **a** and **b**). *p < 0.05, **p < 0.01, ***p < 0.001. The data in **c** were analyzed using one-way ANOVA. The data in **d** were analyzed using paired *t* test. The numbers on the bars refer to the number of coverslips from at least three different batches of cultures

To study the impact of MMP3 excess on GABAergic plasticity we applied exogenous active rMMP-3 and examined NMDA-iLTP in wild-type and *Mmp3*^−/−^ neuronal cultures. Synaptic recordings from cultured hippocampal neurons prepared from *Mmp3*^−/−^ mice indicated that MMP3 deficiency abolished NMDA-induced iLTP (ratio of mIPSC amplitude after/before iLTP; wild type: 1.22 ± 0.05; *Mmp3*^−/−^: 1.01 ± 0.03; *p* = 0.001; Fig. 7a), similar to hippocampal slices (Fig. 2a). We next examined whether exogenous active rMMP3 application together with NMDA (for 2 min 15 s) restores iLTP in *Mmp3*^−/−^ mice. The administration of active rMMP3 during NMDA application restored iLTP in *Mmp3*^−/−^ neurons (*Mmp3*^−/−^ + rMMP3 + NMDA: 1.29 ± 0.07; vs. *Mmp3*^−/−^ from Fig. 7a; *p* < 0.001; Fig. 7b, c). Next, we evaluated the effect of rMMP3 application together with NMDA in wild-type cultures. Stimulation with NMDA induced stable iLTP in wild-type neurons (Fig. 7a), but the simultaneous application of NMDA and rMMP3 in this group for 2 min 15 s did not further increase iLTP; instead, it significantly reduced it (wild-type + rMMP3 + NMDA: 1.06 ± 0.05; *vs*. wild-type NMDA-iLTP form Fig. 7a; *p* = 0.029; Fig. 7b, c). Thus, the ability of *Mmp3*^−/−^ neurons to induce iLTP was restored by exogenous rMMP3, whereas when the exogenous protease was applied together with NMDA in wild-type neurons, an impaired NMDA-iLTP was observed suggesting that excess of MMP3 activity is detrimental for GABAergic plasticity (Fig. 7d). Based on these data, it may be proposed that effective iLTP requires fine-tuned MMP3 activity.

The duration of NMDA application determines the direction of GABAergic plasticity. Moderate postsynaptic Ca^2+^ influx drives iLTP [32], and much stronger NMDA receptor activation results in iLTD [33]. We hypothesized that NMDAR activation that is adequate for iLTP induction in the wild-type group may be insufficient in *Mmp3*^−/−^ neurons. We thus extended the duration of NMDA application from 2 min 15 s to 2 min 45 s, but this longer duration did not result in iLTP induction in wild-type or *Mmp3*^−/−^ neurons (Supplemental Fig. 8a–c). Similarly, the prolonged NMDA stimulation did not rescue the curtailed structural plasticity of the synaptic gephyrin area in the presence of UK-356618 (Supplemental Fig. 8d–f). Thus, iLTP impairment that was caused by MMP3 deficiency cannot be explained by diminished NMDA receptor activation because it was not restored by prolonged NMDA stimulation.

### MMP3 deficiency enhances hippocampus-dependent learning and memory

**Fig. 8:**
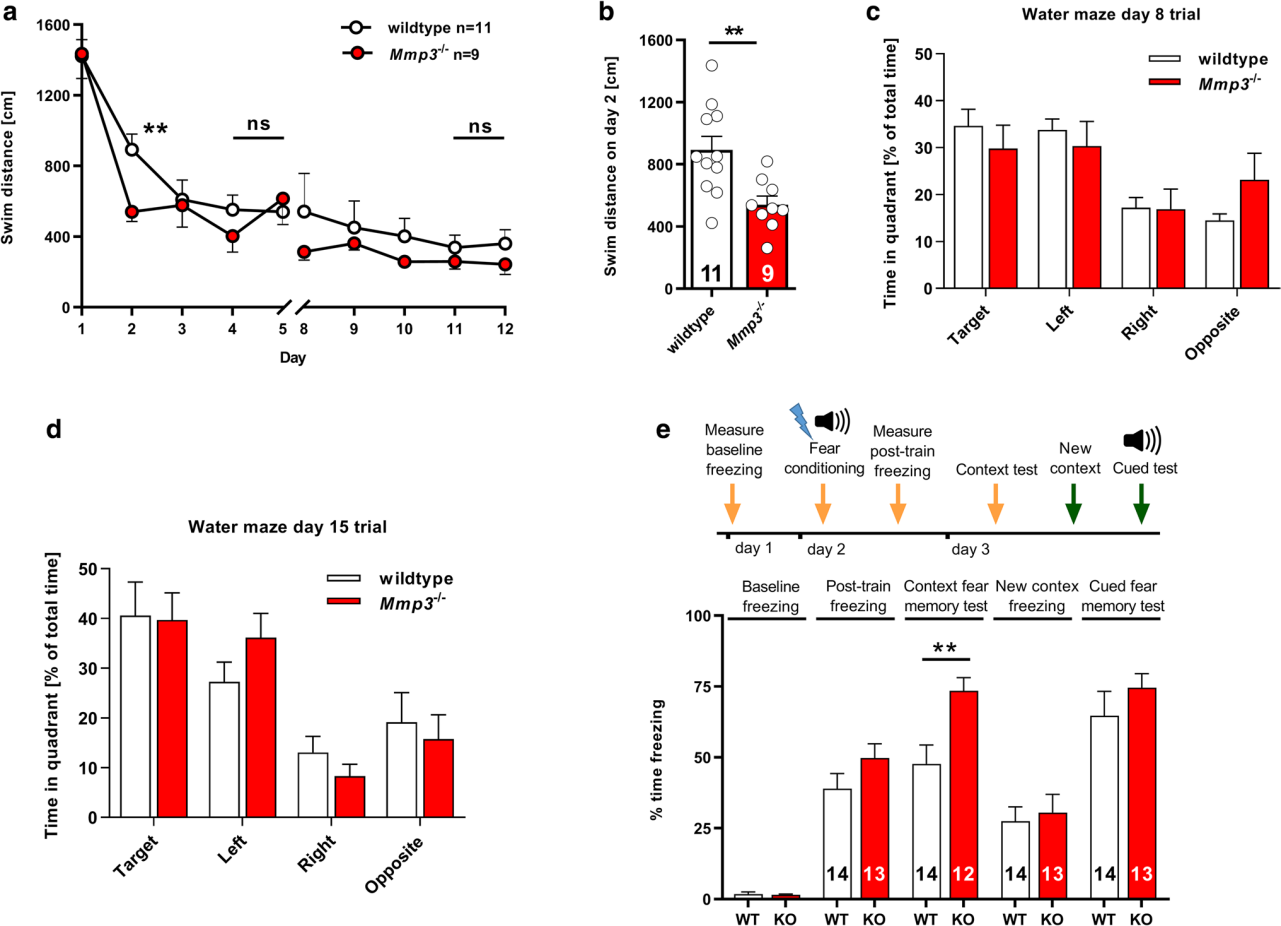
*Mmp3*^−/−^ mice exhibit faster spatial learning and enhanced contextual fear memory. **a**–**d** *Mmp3*^−/−^ mice exhibited a significant difference in spatial learning in the Morris water maze test without changes in memory retrieval. **a** Swim distance to reach the hidden platform during a 10-day learning period. **b** Comparison of swim distances to reach the platform on day 2 between wild-type and *Mmp3*^−/−^ mice. **c**, **d** Percent time spent in each of the quadrants during the probe memory trials on day 8 (**c**) and day 15 (**d**). **e** Experimental design for measurements of contextual and cued fear memory. Different arrow colors indicate distinct experimental cages (spatial contexts). *Mmp3*^−/−^ mice (KO) exhibited a significant enhancement of contextual fear memory 24 h after contextual fear conditioning in comparison to wild-type mice (WT). *Mmp3*^−/−^ mice exhibited no significant changes in basal freezing, freezing during fear conditioning, or freezing in the new context (pre-CS). *Mmp3*^−/−^ mice exhibited similar cue-related memory retention. **p < 0.01. The data in **a**, **c**, **d**, and **e** were analyzed using two-way ANOVA. The data in **b** and **c** for the single comparison between genotypes were analyzed using *t* tests. The numbers on the bars indicate the number of animals

To investigate the possible behavioral role of MMP3 we sought to assess hippocampus-dependent spatial learning and memory in *Mmp3*^−/−^ mice using the Morris water maze. Both *Mmp3*^−/−^ and wild-type animals were trained in four trials per day for 10 days in the hidden-platform version of the Morris water maze. As training progressed, the path length (i.e., a measure of the total distance travelled to reach the platform) decreased in both wild-type and *Mmp3*^−/−^ mice (Fig. 8a). Path length was chosen as a performance measure because it reflects cognitive function in the Morris water maze [34]. The two-way analysis of variance (ANOVA) indicated a significant main effect of genotype on learning curves (*F*_1,180_ = 5.35, *p* = 0.022). Interestingly, in *Mmp3*^−/−^ mice, the path length decreased more rapidly over consecutive trials, suggesting faster learning, reflected by the average day 2 swimming distance to reach the platform (wild type: 892 ± 88 cm; *Mmp3*^−/−^: 541 ± 54 cm; *p* = 0.0047; Fig. 8b). To test spatial reference memory, a probe trial without the platform was performed on days 8 and 15, but no difference in the time spent in the target quadrant was found between genotypes (probe 1: genotype × distance interaction, *F*_3,72_ = 1.29, *p* = 0.29, Fig. 8c; probe 2: genotype × distance interaction, *F*_3,72_ = 0.77, *p* = 0.52; Fig. 8d). Thus, spatial learning in the Morris water maze progressed faster in *Mmp3*^−/−^ mice. After completion of the learning trials, however, *Mmp3*^−/−^ mice had the same spatial reference memory score as wild-type controls.

In the next series of behavioral experiments, we evaluated the contribution of MMP3 to the associative learning of conditioned fear. Within this experiment baseline freezing in the test cage was recorded on day 1 (Fig. 8e). On day 2, footshocks were paired with the spatial context of the test cage and an acoustic signal. Upon returning the animals to the test cage on day 3, memory of the spatial context was examined by analyzing the freezing response. Finally, the context of the test cage was modified to measure freezing in a different spatial context. The mice were tested for cued fear memory by analyzing freezing in response to the acoustic signal. Both wild-type and *Mmp3*^−/−^ mice exhibited similar freezing during habituation and during the acquisition phase of fear conditioning, suggesting the lack of an anxiety-related phenotype and no short-term or working memory deficits. Remarkably, the *Mmp3*^−/−^ mice exhibited a significant increase in freezing during the context-related memory retention test 24 h after learning (wild type: 47 ± 6.7% freezing time; *Mmp3*^−/−^: 73 ± 4.6% freezing time; *p* = 0.0053; Fig. 8e). Additionally, *Mmp3*^−/−^ and wild-type mice exhibited similar freezing in the modified spatial context on day 3 (wild type: 27 ± 5.1% freezing time; *Mmp3*^−/−^: 31 ± 6.4% freezing time; *p* = 0.70) and exhibited a normal freezing response to the acoustic signal, suggesting no changes in amygdala-dependent cued fear conditioning (wild type: 65 ± 8.6% freezing time; *Mmp3*^−/−^: 75 ± 5.0% freezing time; *p* = 0.34; Fig. 8e). These behavioral findings suggest that the lack of MMP3 activity accelerates spatial learning in the Morris water maze and enhances spatial associative fear memory.

## Discussion

In the present study, we found that MMP3-dependent extracellular proteolysis plays a central role in postsynaptic GABAergic plasticity that is induced heterosynaptically by short-lasting NMDA stimulation. Our finding of the crucial role of MMP3 activity in this form of inhibitory plasticity was confirmed both at functional and structural level using various experimental approaches. First, pharmacological inhibition or genetic deficiency of MMP3 completely abolished iLTP. Second, the expression of iLTP was accompanied by a larger area of synaptic gephyrin clusters, whereas this phenomenon was not observed when this induction protocol was applied in the presence of the MMP3 inhibitor or in *Mmp3*^−/−^ neurons. Importantly, the structural reorganization of inhibitory synapses upon iLTP induction has been consistently shown to be a crucial determinant of plastic changes in the present model [29, 35]. MMP3 inactivation affected both functional and structural aspects of postsynaptic iLTP, underscoring the key role of this enzyme and suggesting that it might be involved in molecular mechanisms of iLTP at their early stages. Third, 20 minutes after iLTP induction, α_1_GABA_A_ receptors were characterized by slower and more constrained diffusion within inhibitory synapses. Conversely, when MMP3 was blocked, the lateral movement of GABA_A_ receptors was comparable to that before NMDA application (Fig. 5). Thus, the key mechanism of trapping GABA_A_ receptors within the synaptic densities, whereby iLTP is being built up in this model, critically depends on MMP3 activity. Fourth, the central role of MMP3 in GABAergic plasticity was further demonstrated by the fact that the major manifestations of iLTP (i.e., increase in mIPSC amplitude, enlargement of synaptic gephyrin clusters, and the slower lateral diffusion of synaptic α_1_GABA_A_ receptors) were evoked by exogenous, short (2–3 min) administration of active rMMP3. This observation further suggests that MMP3 is involved in mechanisms of iLTP induction at early stages, which was corroborated by the finding that MMP3 is operative during iLTP induction only within a relatively narrow time window (approximately 10 min). Furthermore, increased area and decreased average fluoresce intensity of gephyrin clusters after rMMP3 application suggest gephyrin dispersal or redistribution within the synapse. Finally, no additive effects of NMDA and MMP3 on the size of gephyrin synaptic clusters were found (Fig. 6). The application of NMDA and rMMP3 together in wild-type neurons weakened functional iLTP (mIPSC amplitude) despite the increase in gephyrin cluster area to the level observed after NMDA-iLTP or MMP3-iLTP. However, while NMDA-iLTP leaved average fluorescence intensity of gephyrin synaptic clusters unchanged, the MMP3-iLTP decreased it. Additionally, exogenous rMMP3 application in *Mmp3*^−/−^ neurons rescued the increase in mIPSC amplitude after iLTP (Fig. 7). These observations provide evidence that MMP3-iLTP at the molecular level belong to the same molecular pathway that underlies the induction of NMDA-iLTP.

Although, to the best of our knowledge, this is the first report demonstrating a key role for MMP3 in GABAergic synaptic plasticity, this enzyme has been previously implicated in glutamatergic synaptic plasticity and related cognitive tasks [22–24]. The involvement of MMP3 in plasticity phenomena is unsurprising because this enzyme is expressed in an activity-dependent manner in the developing and mature brain, where it operates mainly in the extracellular space in the vicinity of synapses [20, 36]. Importantly, for glutamatergic synapses, the activity-dependent release and short-term activation of extracellular proteases allow the precise cleavage of adhesion proteins and extracellular matrix (ECM) proteoglycans that, in turn, modify the local milieu of excitatory synapses [11]. However, discernment of the molecular mechanisms whereby MMP3 regulates synaptic signaling is complicated by the rich repertoire of putative MMP substrates [36]. Several potential substrates for MMP3 can be recruited among proteins and glycosaminoglycans of the synaptic adhesive apparatus and brain ECM that are involved in regulating synaptic structure and function [37]. MMP3 can cleave brain proteoglycans, collagens, laminin, tenascins, and extracellular signaling proteins (e.g., pro-brain-derived neurotrophic factor, plasminogen, and cytokines) [36]. Notably, Conant et al. [38] reported that glutamatergic LTP was associated with the MMP3-dependent shedding of intercellular adhesion molecule-5. Whether the cleavage of these substrates is relevant to GABAergic plasticity remains to be elucidated.

In general, the ECM has at least two macroscopic forms: (i) condensed perineuronal nets (PNNs) that enwrap the soma of parvalbumin-positive interneurons and a subset of pyramidal cells and (ii) a diffuse perisynaptic matrix that constitutes > 90% of the brain ECM [39]. Both forms of ECM, together with the synaptic adhesive apparatus, was proposed to stabilize synaptic structures, and ECM modification through proteolysis may make synapses susceptible to plastic changes and engram encoding [40]. The presence of PNNs limits neuroplasticity, and the disruption of these structures may at least partially relieve this constraint. For example, Pizzorusso et al. [41] reported that the hydrolysis of chondroitin sulfate proteoglycans reactivated ocular dominance plasticity in the adult visual cortex. Similarly, intact PNNs in the amygdala protect fear memories from erasure, because the maturation of perineuronal nets underlie the molecular mechanism that closes a postnatal critical period during which traumatic memories can be erased through an extinction mechanism [42]. However, chondroitinase ABC usually used to hydrolyze PNNs is exogenous and has no endogenous counterpart in the mammalian brain. Future studies should determine whether MMP3 could be an endogenous factor involved in shaping PNNs during GABAergic plasticity and thereby affecting memory formation. This possibility is particularly interesting because several PNN constituents are substrates for MMP3 [36]. Finally, the most prominent PNNs are present on parvalbumin-positive GABAergic interneurons but it is unknown whether these structures affect GABAergic synaptic plasticity.

Several lines of evidence indicate that MMP3 favors iLTP in the present model. There are numerous ways in which the activity of extracellular proteases translates into alterations of the diffusion and synaptic trapping of neurotransmitter receptors. At excitatory synapses, hydrolysis of the brain ECM by hyaluronidase or chondroitinase ABC was proposed to remove “hurdles” that restrict the lateral diffusion of AMPA receptors, thus allowing the efficient exchange of desensitized receptors to naive ones [43]. Similarly, MMP9, through the cleavage of an unknown substrate, activates integrin signaling that, in turn, increases NMDA receptor mobility in both synaptic and extrasynaptic membranes [44]. Intriguingly, an opposite scenario for GABA_A_ receptors was observed in the present study, in which the administration of active MMP3 slowed the diffusion of only synaptic receptors and favored their trapping. Most likely, in the case of iLTP, we are dealing with a more complex signaling which does not rely merely on affecting diffusion by altering the physical environment of GABA_A_ receptors. However, the exact molecular mechanism of the plasticity that was observed herein remains to be determined.

A precise balance between inhibition and excitation in neuronal networks must be assured by the fine-tuning of inhibitory connections [4, 45], which can be achieved by various forms of plasticity that occur at different inhibitory synapses. The present results suggest that the lack of MMP3 activity, which results in impairments in iLTP, enhances hippocampus-dependent spatial learning. This possibility is unsurprising because both proteolysis and the modulation of GABAergic drive are known to affect various forms of learning and memory formation. For example, the enzymatic degradation of glycosaminoglycans by chondroitinase ABC prolonged object recognition memory [46] and the digestion of brain hyaluronan enhanced cognitive flexibility in gerbils [47]. However, MMP3 deficiency  reduces ECM digestion; therefore, the cognitive enhancement that was observed in the present study cannot be ascribed to extensive ECM cleavage. Although anatomical studies ruled out gross morphological changes in CA1 pyramidal neurons of *Mmp3*^−/−^ mice [48], as well as an impairment in NMDA-dependent excitatory LTP [20], it cannot be excluded at the present stage that compensatory changes in the genetically modified animals could have contributed to the observed behavioral phenotype in this model.

Various factors that alter GABAergic inhibition are known to impinge cognition. For example, the genetic knockout of α4 or α5 subunit of GABA_A_ receptors improved spatial learning in the Morris water maze and contextual fear conditioning [49, 50]. Similarly, a pharmacological decrease in GABAergic transmission facilitated the acquisition of fear memory and memory retention in passive avoidance learning [51, 52]. Furthermore, the chemogenetic silencing of somatostatin-expressing interneurons during learning was reported to increase the number of neurons that were recruited to the engram and enhanced contextual fear conditioning [53]. In the present study, *Mmp3* knockout prevented iLTP, which might also be regarded as a factor debilitating the inhibition. The augmentation of fear conditioning in *Mmp3*^−/−^ mice may thus be explained by the lack of GABAergic iLTP, but the direct causal link between GABAergic plasticity and alterations of learning requires further studies. In addition, several recent studies have shown that learning is accompanied by plastic changes at GABAergic synapses that constrain memory recall [54]. The lack of such plasticity may be expected to increase memory recall, as observed in *Mmp3*^−/−^ mice. Nevertheless, while MMP3 is known to contribute to hippocampal-dependent learning, at the present stage, our findings concerning GABAergic plasticity and memory should be regarded as correlative at best, while causative mechanisms still need to be identified.

In conclusion, we provide the first evidence that iLTP strongly relies on MMP3 activity beginning from the early phases of plasticity induction. The present findings also suggest that impairments in NMDA-induced iLTP during learning may enhance memory, but a direct causal link between GABAergic plasticity and learning awaits further investigation.

## Materials and methods

### Animals

The animals of either sex were housed on a natural light/dark (12 h/12 h) cycle and received food and water ad libitum. All of the experiments were performed in accordance with the guidelines of the European Communities Council and approved by the Local Bioethical Committee for Experiments on Laboratory Animals.

### Primary hippocampal cultures

Primary hippocampal cell cultures were prepared from postnatal day 0 (P0) to P2 C57BL/6 J wild-type and *Mmp3*^−/−^ mouse pups [55]. Neurons were plated on poly-l-lysine (for single-particle tracking, Sigma-Aldrich) or laminin (Roche) and poly-l-lysine-coated 18 mm-diameter coverslips at a density 2.0 × 10^4^ cells/cm^2^ for immunolabeling and 3.1 × 10^4^ cells/cm^2^ for electrophysiological recordings. Neuronal cultures were kept in Neurobasal-A medium (Gibco) supplemented with B-27 (1:100; Gibco) at 37 °C in 5% CO_2_. On 2–3 days in vitro (DIV), half of the medium was exchanged for Neurobasal-A/B-27 with Ara-C (25 μM, Sigma-Aldrich). The experiments were performed on cells that were cultured for 12–19 days.

### Slice preparation

Hippocampal slices (350 μm) were prepared from 18- to 21-day-old C57BL/6 wild-type or *Mmp3*^−/−^ mice. After decapitation, the brains were immersed in cold artificial cerebrospinal fluid (aCSF; 119 mM NaCl, 26.3 mM NaHCO_3_, 11 mM glucose, 2.5 mM KCl, 1 mM NaH_2_PO_4_, 1.3 mM MgSO_4_, and 2.5 mM CaCl_2_, pH 7.4) that was saturated with carbogen (95% O_2_, 5% CO_2_) and cut using a vibrating microtome (Leica VT1200S). After sectioning, the slices were transferred to a recovery chamber that contained the same aCSF at room temperature.

### Electrophysiological recordings

All of the electrophysiological recordings were performed in aCSF, using borosilicate patch pipettes that were filled with an intracellular solution that contained the following: 10 mM potassium gluconate, 125 mM KCl, 1 mM EGTA, 10 mM HEPES, 4 mM MgATP, and 5 mM sucrose, pH 7.25, 295 MOsm [25]. In slices, after at least a 1-h recovery period, we performed whole-cell patch-clamp recordings of GABA-mediated postsynaptic mIPSCs from hippocampal CA1 pyramidal neurons. Pyramidal cells were first identified visually under a high-power water immersion objective (40× magnification) with infrared differential interference contrast. In the slices, pyramidal cells were also distinguished from interneurons based on their firing pattern consisting of regular train of action potentials and prominent sag occurrence. Baseline mIPSC measurements were recorded for at least 20 min in the presence of selective blockers of non-NMDA glutamate receptors (20 μM DNQX) and Na^+^ channels (1 μM tetrodotoxin) at a holding potential of − 70 mV. The stability of recordings was checked by monitoring the input resistance during the whole experiment. Cells exhibiting more than 20% changes were excluded from the analysis. Series resistance was not compensated. Currents were digitized at 20 kHz and filtered at 10 kHz using the MultiClamp 700B amplifier and Axon Digidata 1550 (Molecular Devices). mIPSCs were analyzed manually using pClamp10 software (Molecular Devices).

After stable baseline recordings, we induced inhibitory long-term potentiation (iLTP) by transient exposure to NMDA (3 min in slices, 2 min 15 s in cultures, 20 µM) [25] in wild-type and *Mmp3*^−/−^ mice. The agonist was then washed out, and mIPSCs were monitored for at least 30 min. The data were binned into 2-min time bins and then averaged to mean amplitude or frequency values at a given time point. The extent of iLTP was defined as the ratio of the mean amplitude of mIPSC recorded 20–22 min after NMDA application to the amplitude recorded before plasticity induction. The mIPSC rise phase was estimated at 10% to 90% rise time, and the decay phase was fitted with a biexponential function:

$$y(t) = A_{1} \exp \left( - \frac{t}{\tau_{\text{fast}}}\right) + A_{2} \exp \left( - \frac{t}{\tau_{\text{slow}}} \right),$$

where $\tau_{\mathrm{fast}}$ and ${\tau }_{\mathrm{slow}}$ are the time constants, and *A*_1_ and *A*_2_ are the amplitudes of the fast and slow function components, respectively. The mean decay time constant (*τ*_mean_) was calculated as *τ*_mean_ = *a*_1_*τ*_fast_ + *a*_2_*τ*_slow_, where *a*_1_ = *A*1/(*A*1 + *A*2) and *a*_2_ = *A*2/(*A*1 + *A*2).

### Immunofluorescence staining

Cells were fixed in a methanol:acetone (1:1) solution for 20 min at − 20 °C and then rinsed three times with phosphate-buffered saline (PBS; pH 7.4). Immunolabeling with primary antibodies was preceded by membrane permeabilization (0.2% Triton X-100 for 12 min) and incubation with 5% bovine serum albumin (BSA; Santa Cruz Biotechnology) in PBS for 30 min. Reactions with primary antibodies with 1% BSA in PBS were run for 90 min at room temperature. Following three washes (1% BSA in PBS), secondary antibodies with 1% BSA in PBS were applied for 60 min at room temperature. After incubation with primary and secondary antibodies, the cells were washed with 1% BSA in PBS and mounted with Fluoroshield (Sigma-Aldrich).

### Confocal imaging and immunofluorescence analysis

Cells were visualized using an Olympus Fluoview 1000S laser scanning confocal microscope (Olympus, Japan). Images were acquired in the sequential mode as a z-stack with three sections separated by 0.5 µm using a 60× oil immersion objective (PlanApo 1.35) with 473- and 635-nm excitation lasers. On each coverslip, two images from different neurons were analyzed, and the data were averaged. The full analysis of confocal images was performed using ImageJ software (National Institutes of Health and LOCI, University of Wisconsin). Gephyrin clusters were defined as synaptic if they were located within the area of vesicular GABA transporter (vGAT)-positive clusters enlarged by 2 pixels in every direction.

### Single-particle tracking imaging of quantum dot-tagged GABAA receptors

Before quantum dot (QD) labeling, neuronal cell cultures were incubated with anti-vGAT-Oyster550 antibody (Synaptic System) diluted in culture medium at 37 °C for 20 min to visualize GABAergic synapses. Antibody specificity was validated previously [30]. Before QD labeling, rabbit antibody directed against an extracellular epitope of α1-subunit of the GABA_A_ receptor (AGA-001; Alomone) was incubated for 30 min with anti-rabbit QD 655 (Invitrogen) in the presence of casein (Vectorlab). The neurons were then incubated with the diluted antibody–QD complex for 2 min at room temperature. The concentration of the antibody–QD complex was adjusted to elicit the staining of ~ 30 receptors in the field of view to observe trajectories that in majority are not overlapping. Live-cell imaging was performed at 20 Hz using a wide-field inverted microscope (Eclipse Ti, Nikon) with a 60× oil objective (NA 1.4), diode-based illumination (Lumencor, SpectraX Light Engine, Optoprim), an EM-CCD camera (9100, Hamamatsu), and Metamorph 7.8 software (Molecular Devices). Band-pass excitation filters (543/22; 435/40) and emission filters (593/40; 655/15) were used to image Oyster550 and QD-655, respectively. Imaging was performed at 32 °C in a chamber that was continuously perfused with recording solution (145 mM NaCl, 2 mM KCl, 2 mM CaCl_2_, 2 mM MgCl_2_, 10 mM glucose, 10 μM D-serine, and 10 mM HEPES, pH 7.4).

The spatial coordinates of QD were determined using MIA software based on simulated annealing algorithm [56]. For a single QD, the coordinates of successive frames were track-connected using custom MATLAB software, which allowed for of displacement of not more than 4 pixels during the maximal allowable dark period between QD blinks (25 frames). If trajectories of two nearby QDs crossed, then both were rejected from further analysis. The mean square displacement (MSD) for a single trajectory was calculated using the following formula:

$${\text{MSD }}\left( {n{\text{d}}t} \right) \, = \, \left( {N - n} \right)^{ - 1} \Sigma_{i = 1}^{N - n} \left[ {\left( {x_{i + n} {-} \, x_{i} } \right)^{2} + \, \left( {y_{i + n} {-} \, y_{i} } \right)^{2} } \right],$$

where *x*_*i*_ and *y*_*i*_ are the spatial coordinates of a single QD in frame *i*, *N* is the total number of points in the trajectory, and d*t* is the time interval between successive frames (50 ms). From the MSD(*t*) plot, the instantaneous diffusion coefficient, *D*, was calculated as the slope of the linear function MSD(*t*) = 4Dt fitted to the first 2–4 points. During analysis, QD trajectories were considered synaptic when they colocalized with vGAT-positive GABAergic terminals enlarged by 2 pixels in every direction. The immobile fraction parameter was defined as the relative duration of the residence of a receptor–QD complex in a given compartment with coefficient < 0.0075 µm^2^s^−1^. This value has been empirically identified as the minimum of the bimodal distribution of log (*D*) function, which reveals the existence of two receptor-QD populations, one mobile and one poorly mobile (immobile). The residence time was calculated as a ratio of the total time spent by QDs at the synapse to the imaging time (1 min). The MSD vs. time curves were compared using steady-state values and Mann–Whitney *U* test.

### Infusion of NMDA and rMMP3

In pyramidal neurons, moderate postsynaptic Ca^2+^ influx through NMDA receptors usually drives iLTP [30], whereas stronger and longer NMDA receptor activation results in the induction of iLTD [33]. The occurrence of GABAergic plasticity is thus determined by the duration of NMDA application and the solution exchange time. All of our electrophysiological recordings were performed in a submerged chamber on an upright microscope, with continuous perfusion with the recording solution at 2.5 ml/min. Under these conditions, we found that iLTP was effectively induced in neuronal cultures by NMDA application (using perfusion pump) for 2 min 15 s. However, in hippocampal slices, the duration of NMDA application needed to be extended to 3 min (Table 1). This difference [25] may be attributable to a longer solution exchange time in slices compared with cell cultures.

**Table 1:** Duration of treatments used to induce NMDA-iLTP and MMP3-iLTP in different experimental conditions

Substance application	Brain slices electrophysiology (upright microscope)	Neuronal culture electrophysiology (upright microscope)	Neuronal culture immunolabeling (12-well plate)	Neuronal culture SMT (inverted microscope)
Application of NMDA to induce NMDA-iLTP	3 min (Fig. 1,2,3)	2 min 15 s (Fig. 4a–c; 7a,c–d)	2 min 15 s (Fig. 4d–g)	2 min (Fig. 5)
Application of rMMP3 to induce MMP3-iLTP	–	3 min (Fig. 6a–d)	2 min 15 s (Fig. 6i, j)	2 min (Fig. 6e–h)
Application of NMDA with rMMP3	–	2 min 15 s (Fig. 7b–d)	2 min 15 s (Fig. 6k, l)	–
Prolonged application of NMDA	–	2 min 45 s (Suppl Fig. 8a–c)	2 min 45 s (Suppl Fig. 8d–f)	–

In the experiments with exogenous rMMP3, we applied recombinant protein for different times, depending on the experimental setup. During single-molecule tracking that was performed on an inverted microscope, we acutely treated neuronal cultures with active rMMP3 for 2 min and exchanged the solution swiftly by pipetting. During electrophysiological recordings of neuronal cultures, we applied rMMP3 through the perfusion pump for 3 min because shorter times gave inconsistent results that were likely attributable to constrained solution exchange that was caused by the presence of an upright microscope objective in the recording chamber. Additionally, during the gephyrin immunolabeling experiments, we incubated neuronal cultures with rMMP3 for 2 min 15 s to match the duration of iLTP inducing application of NMDA.

### Morris water maze

Spatial learning ability was tested in a typical hidden-platform Morris water maze (circular pool, 150 cm diameter) that was filled with 25–26 °C water that was made opaque with nontoxic white paint as previously described [57, 58]. The mice were trained to find the platform during two blocks of five consecutive daily acquisition sessions, followed by 2 days of rest. Each session consisted of four swimming trials, with a 15-min intertrial interval. Each trial began at one of four starting locations that were randomly selected. The mice were guided to the platform if they failed to find it within 2 min and remained on the platform for 15 s. The platform remained at a fixed position throughout the learning trials but was removed from the pool during the probe trials. To evaluate retention memory, probe trials were performed on days 8 and 15, during which the mice were allowed to swim freely for 100 s. Swimming paths were recorded using Ethovision equipment and software (Noldus).

### Contextual/cued fear conditioning

The contextual fear conditioning experiment was performed as previously described [58, 59]. On day 1, the mice were placed in the testing chamber (dark Plexiglas cage with a grid floor) and allowed to habituate to it for 5 min. On day 2, the mice were placed in the testing chamber. After a 2-min baseline exploration period, a tone cue (4 kHz, 80 dB) that served as the conditioned stimulus (CS) was delivered for 30 s, which co-terminated with a 2-s mild footshock (0.3 mA) that served as the unconditioned stimulus (US). After 60 s, this CS-US pairing was repeated, followed by 30 s of exploration. On day 3, memory retention of either the context or tone was assessed. At first, the mice were placed in the cage for 5 min without CS or US presentation (context test). After 2–3 h, the mice were placed in a new context (no grid floor, lights on, and mint odor) for 3 min (pre-CS period), and then a 3-min tone was presented without the US (CS test period). The percent time spent freezing was recorded and analyzed for each time period in the various experimental sessions. See Fig. 8e for a schematic overview.

### Drugs and antibodies

We used the following MMP inhibitors: FN-439 (180 μM, Calbiochem), UK-356618 (2 μM, Sigma-Aldrich), and SB3-CT (10 μM, Sigma-Aldrich). FN-439 is a broad-spectrum MMP inhibitor that inhibits MMP1, MMP2, MMP3, MMP8, and MMP9 at the tested concentration. UK-356618 inhibits the activity of MMP3 and MMP13 at 2 µM and partially blocks MMP9 (*K*_*i*_ for MMP9 = 840 nM). SB-3CT is a specific inhibitor of MMP2 and MMP9 (*K*_*i*_ for MMP2 = 14 nM; *K*_*i*_ for MMP9 = 600 nM) [20]. Recombinant human active rMMP3 (Sigma, catalog no. SRP7783) was applied at a concentration of 400 ng/ml.

Anti-gephyrin (catalog no. 147 111, 1:500) and anti-vGAT (catalog no. 131 006, 1:500) antibodies were purchased from Synaptic System. Alexa 488 and Alexa 633 fluorescent secondary antibodies against mouse and chicken antibodies were obtained from ThermoFisher Scientific.

### Statistical analysis

The statistical analyses of data were performed using SigmaPlot (Systat Software) and GraphPad Prism 8. The electrophysiological and immunocytochemical data are expressed as mean ± SEM; the diffusion coefficients measured using single-particle tracking approach are expressed as median ± interquartile range (IQR 25–75%). Datasets with a normal distribution were compared using two-tailed Student’s *t* test (paired or unpaired), one-way or two-way analysis of variance (ANOVA) with Bonferroni correction. Datasets with a non-Gaussian distribution were analyzed using the nonparametric two-tailed Mann–Whitney *U* test (unpaired comparisons) or Wilcoxon signed-rank test (paired comparisons). All statistical tests were two-tailed and were specified in figure legends. The sample sizes are specified in the graphs. For the cell culture electrophysiological and morphological experiments, *n* refers to the number of coverslips with neurons from at least three different preparations. Values of *p* < 0.05 were considered statistically significant.

## Electronic supplementary material

Below is the link to the electronic supplementary material.

Supplementary file1 (DOCX 1771 kb)
