# Supplementary material for: Long-term plasticity of inhibitory synapses in the hippocampus and spatial learning depends on matrix metalloproteinase 3
Source: Cell Mol Life Sci. 2020 Sep 21;78(5):2279–98. doi: 10.1007/s00018-020-03640-6 (PMC7966195; doi:10.1007/s00018-020-03640-6)
Supplement: Supplementary file 1 — Supplementary file1 (DOCX 1771 kb) [file 18_2020_3640_MOESM1_ESM.docx]

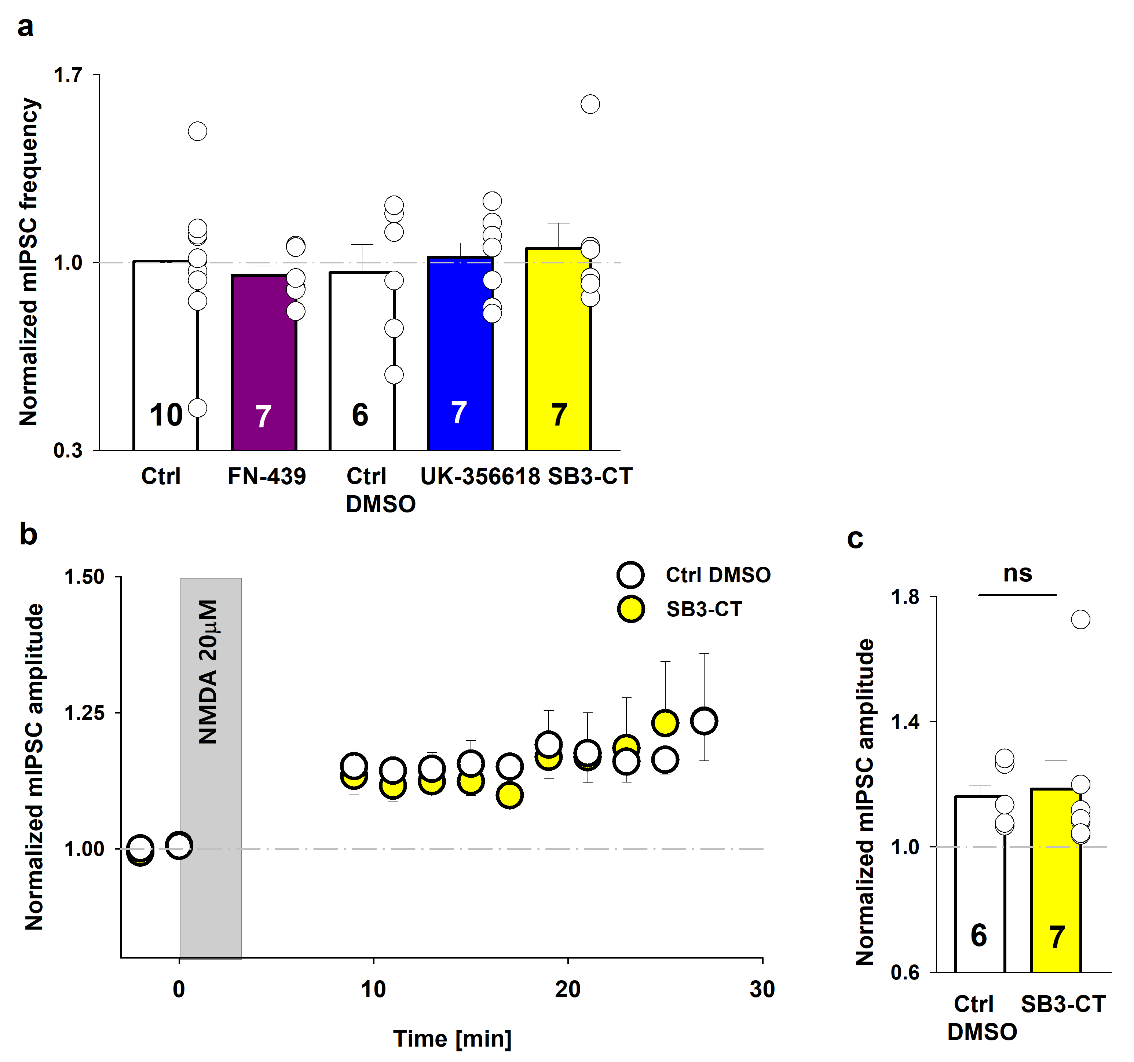


**Supplementary Fig. 1. Gelatinase activity is not needed for iLTP induction in hippocampal slices.**

**(a)** Summary of normalized mIPSC frequency (measured at 20-22 min after iLTP induction relative to values before NMDA treatment) in control conditions (NMDA treatment only – Ctrl, NMDA stimulation in the presence of DMSO – DMSO; white bars) and when MMP inhibitors were applied (FN-439, UK-356618 and SB-3CT). Note lack of any significant changes between analysed groups.

**(b)** Time course of relative mIPSC amplitude after iLTP induction, recorded from control slices (white circles) and in the presence of SB-3CT (yellow circles). The gray area marks the application of NMDA.

**(c)** Statistics for iLTP magnitude (a ratio of mIPSC amplitude measured 20-22 min after iLTP induction to baseline amplitude) in control and SB-3CT-treated slices.

ns - nonsigniﬁcant; One Way ANOVA (**a**), unpaired *t*-test (**c**). The numbers in the bars refer to the number of recordings.

**
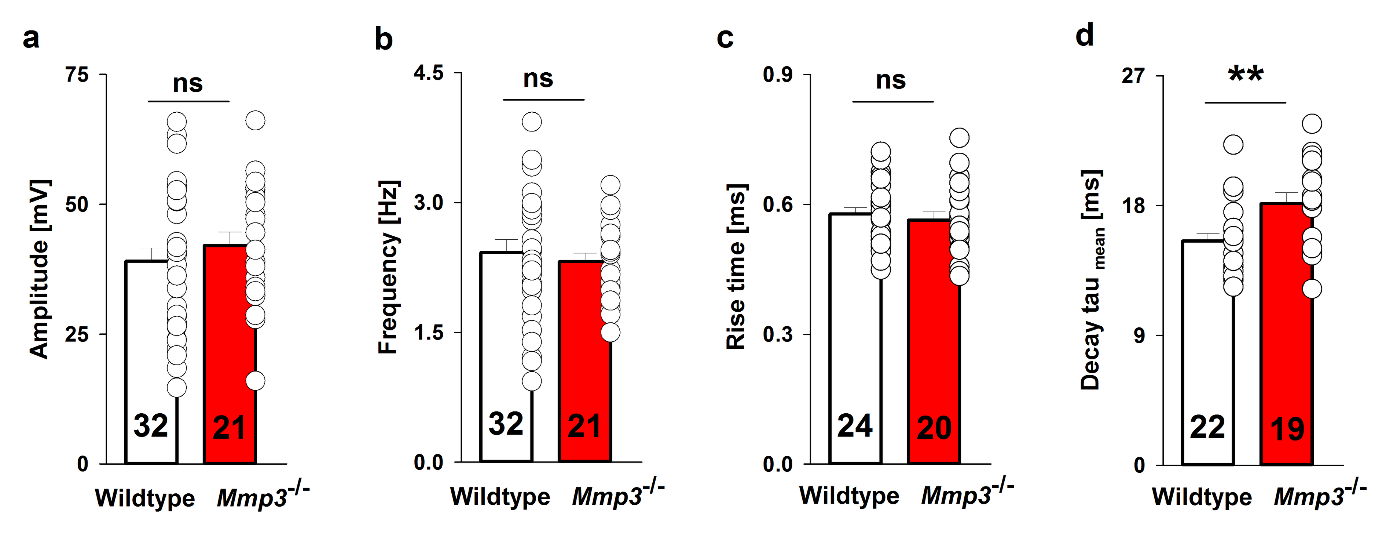
**

**Supplementary Fig. 2. Properties of mIPSCs recorded from pyramidal neurons in wildtype and *Mmp3*^-/-^ slices.**

**(a-d)** Comparison of basic mIPSC properties in wildtype (white bars) and *Mmp3*^-/-^ (red bars) brain slices; (a) amplitude (unpaired *t*-test), (b) frequency (Mann-Whitney U-test), (c) rise time (unpaired *t*-test), (d) decay τ_mean_ (unpaired *t*-test).

ns - nonsignificant; **p < 0.01. The numbers in the bars refer to the number of recordings.

**
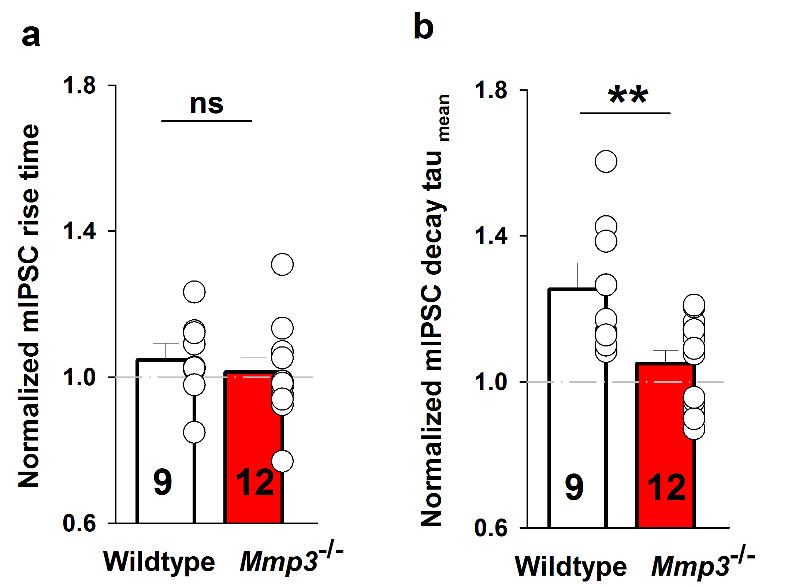
**

**Supplementary Fig. 3. Changes in mIPSC kinetics after iLTP induction.**

Mean mIPSC rise time (**a**) and decay τ_mean_ (**b**) after NMDA stimulation are plotted relative to the mean rise time (unpaired *t*-test) and decay τ_mean_ (unpaired *t*-test) before treatment. Note that the mIPSC decay phase was affected by NMDA stimulation in wildtype brain slices, but not in *Mmp3*^-/-^ slices.

***p* < 0.01, ns, non signiﬁcant. The numbers in the bars refer to the number of recordings.

**
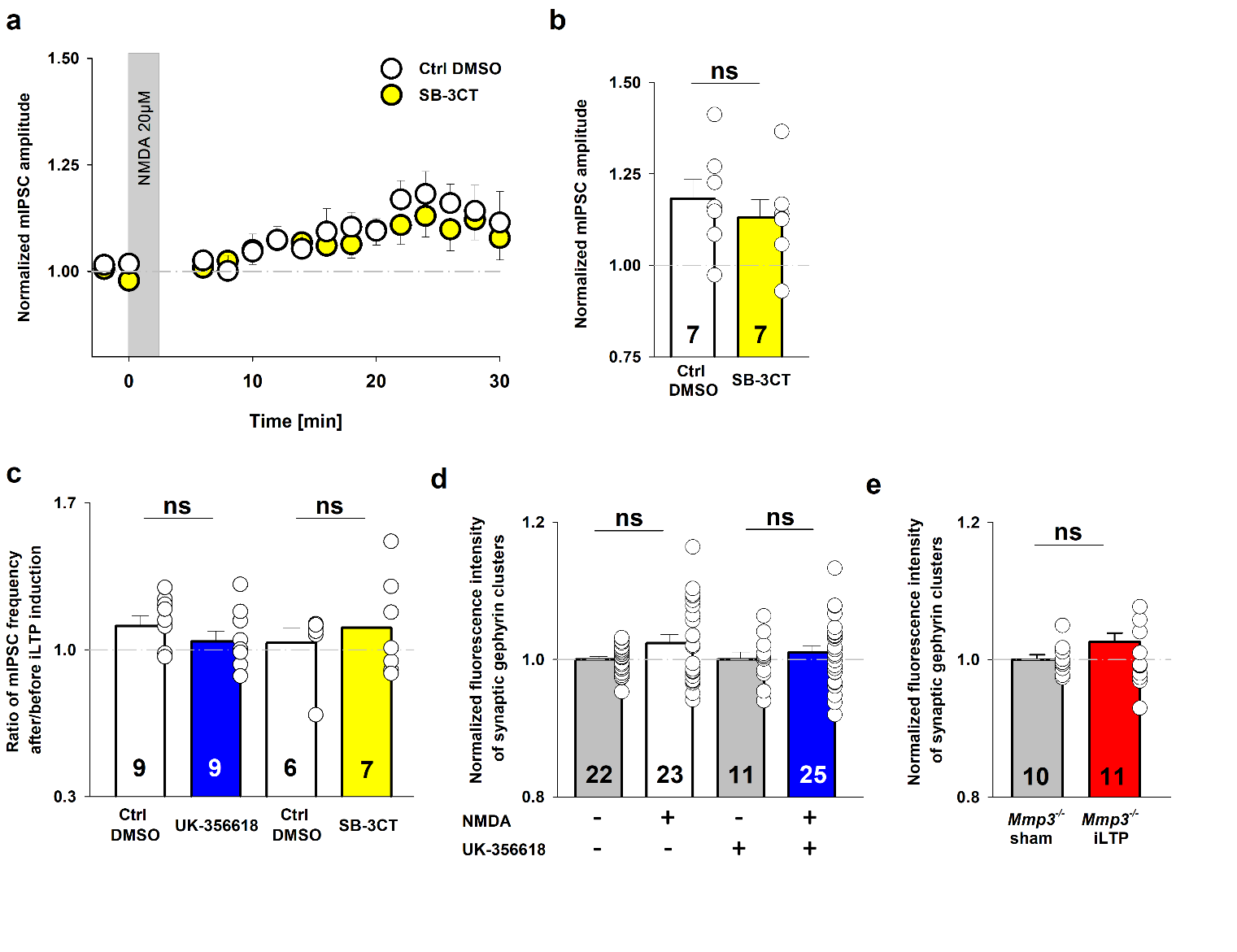
**

**Supplementary Fig. 4 MMP2/9 are not involved in iLTP induction in neuronal cultures.**

1. mIPSC amplitude recorded in neuronal cultures in control group (presence of DMSO) and incubated with MMP2/9 inhibitor SB-3CT (10µM). The gray area marks the application of NMDA.
2. Comparison of changes in mIPSC amplitude when iLTP was induced in control group or in the presence of SB-3CT.
3. Comparison of putative changes in mIPSC frequency after iLTP induction. Every bar represents the ratio of mIPSC frequency recorded 20-22 min after iLTP induction to frequency recorded before induction.
4. Quantification of the normalized total fluorescence intensity of synaptic gephyrin clusters in neuronal cultures treated with respective vehicle (gray), NMDA (iLTP, white), and NMDA with UK-356618 (blue).
5. Quantification of the normalized total fluorescence intensity of synaptic gephyrin clusters in *Mmp3^-/-^* neurons in response to sham solution (gray) and NMDA (red).

ns - nonsigniﬁcant; t-test vs. respective control group (b-e). The numbers in the bars refer to the number of recordings.

**
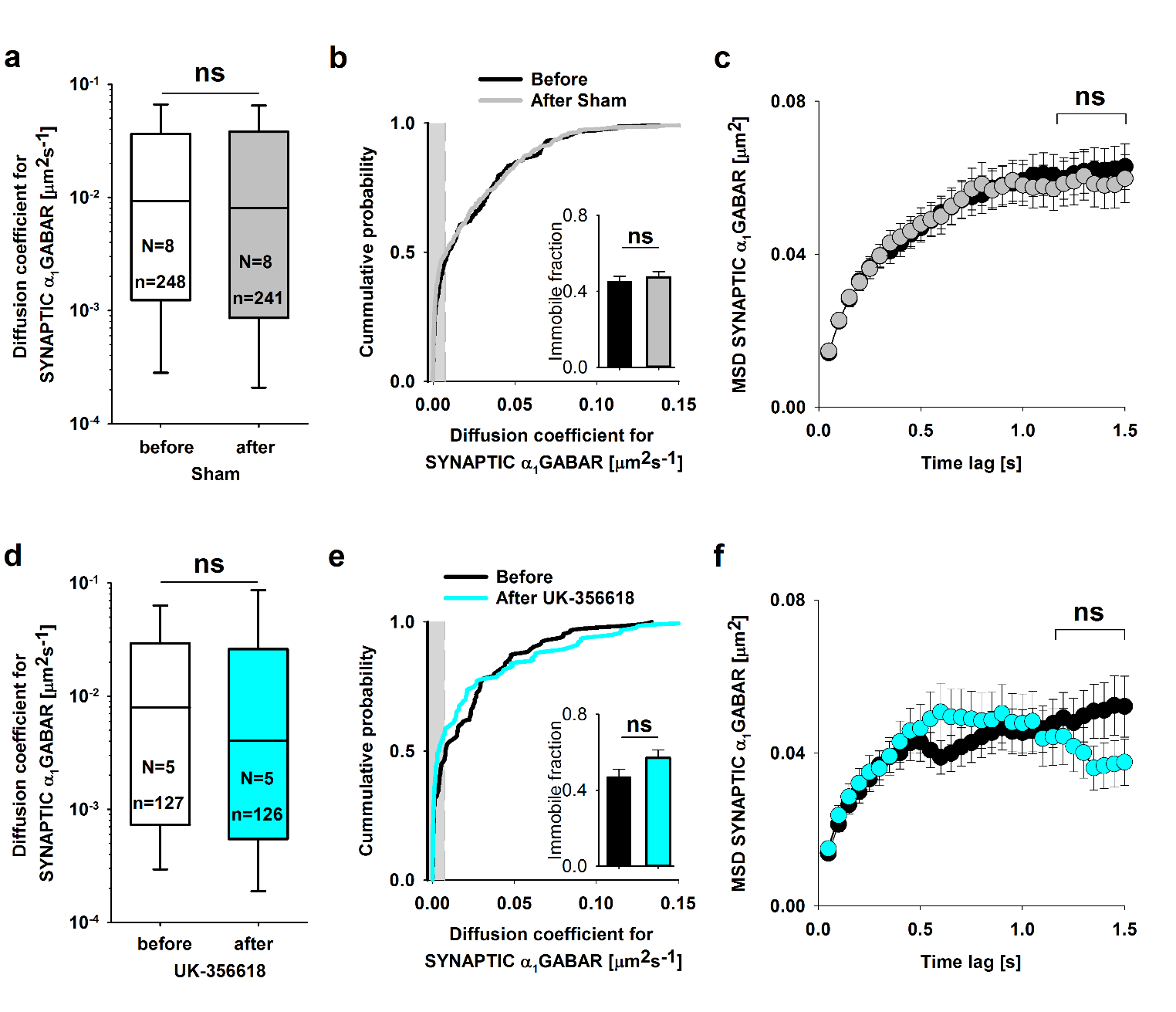
**

**Supplementary Fig. 5. Neither sham treatment nor the presence of UK-356618 affects lateral diffusion of synaptic α_1_GABA_A_ receptors.**

1. Interquartile range (IQR; 25-75%) and median diffusion coefficient of synaptic α_1_GABA_A_ receptors before and 20 min after sham stimulation with vehicle. N and n refer to the number of coverslips and analyzed trajectories, respectively.
2. Cumulative probability distributions of diffusion coefficients for synaptic α_1_GABA_A_ receptors before (black) and 20 min after sham stimulation (gray). The gray area marks the part of the cumulative distribution that contains immobile receptors (D < 0.0075 µm^2^s^-1^). (**Inset**) comparison of the immobile fraction before and after sham treatment.
3. Mean square displacement of synaptic α_1_GABA_A_ receptors before (black) and 20 min after sham stimulation (gray).
4. Interquartile range (IQR; 25-75%) and median diffusion coefficient of synaptic α_1_GABA_A_ receptors before and 20 min after superfusion of UK356618. N and n refer to the number of coverslips and analyzed trajectories, respectively.
5. Cumulative probability distributions of diffusion coefficients for synaptic α_1_GABA_A_ receptors before (black) and 20 min after start of UK-356618 superfusion (cyan). The gray area marks the part of the cumulative distribution that contains immobile receptors (D < 0.0075 µm^2^s^-1^). (**Inset**) comparison of the immobile fraction before and after UK-356618 treatment.
6. Mean square displacement of synaptic α_1_GABA_A_ receptors before (black) and 20 min after UK-356618 superfusion (cyan).

ns - nonsigniﬁcant; Mann–Whitney U test (**a, b - inset**, **c**, **d, e – inset, f**). . N and n refer to the number of coverslips and analyzed trajectories, respectively

**
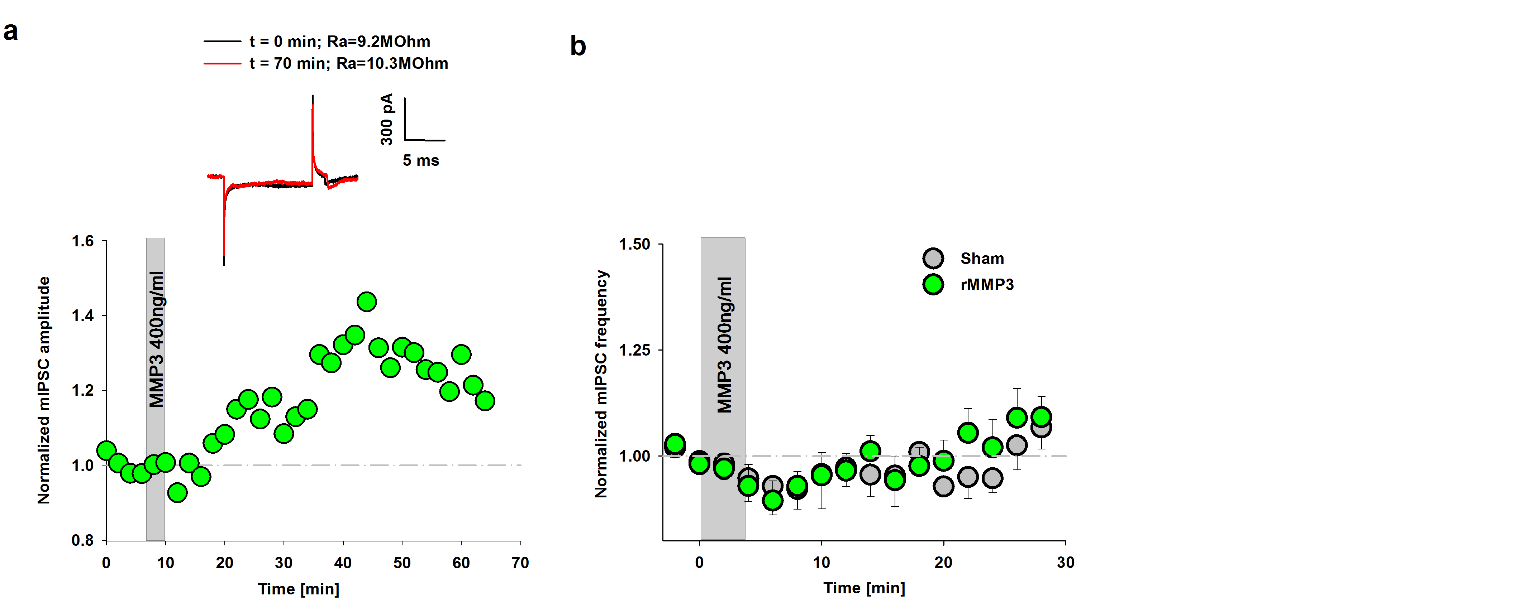
**

**Supplementary Fig. 6. Short-term application of exogenous active rMMP3 increases mIPSC amplitude but not frequency.**

1. mIPSC amplitudes recorded over more than one hour after application of recombinant active MMP3 (400 ng/ml) for 3 min at t = 8 min (gray area). Insets above contain responses to depolarization step recorded before rMMP3 application (black) and 1h after (red). Access resistance of the seal is indicated.
2. mIPSC frequency recorded in sham-treated group (gray) and in cell cultures incubated for 3 min with recombinant active MMP3 (400 ng/ml; green). Comparison of mIPSC frequency recorded after sham and rMMP3 treatment show no significant difference (*t*-test).


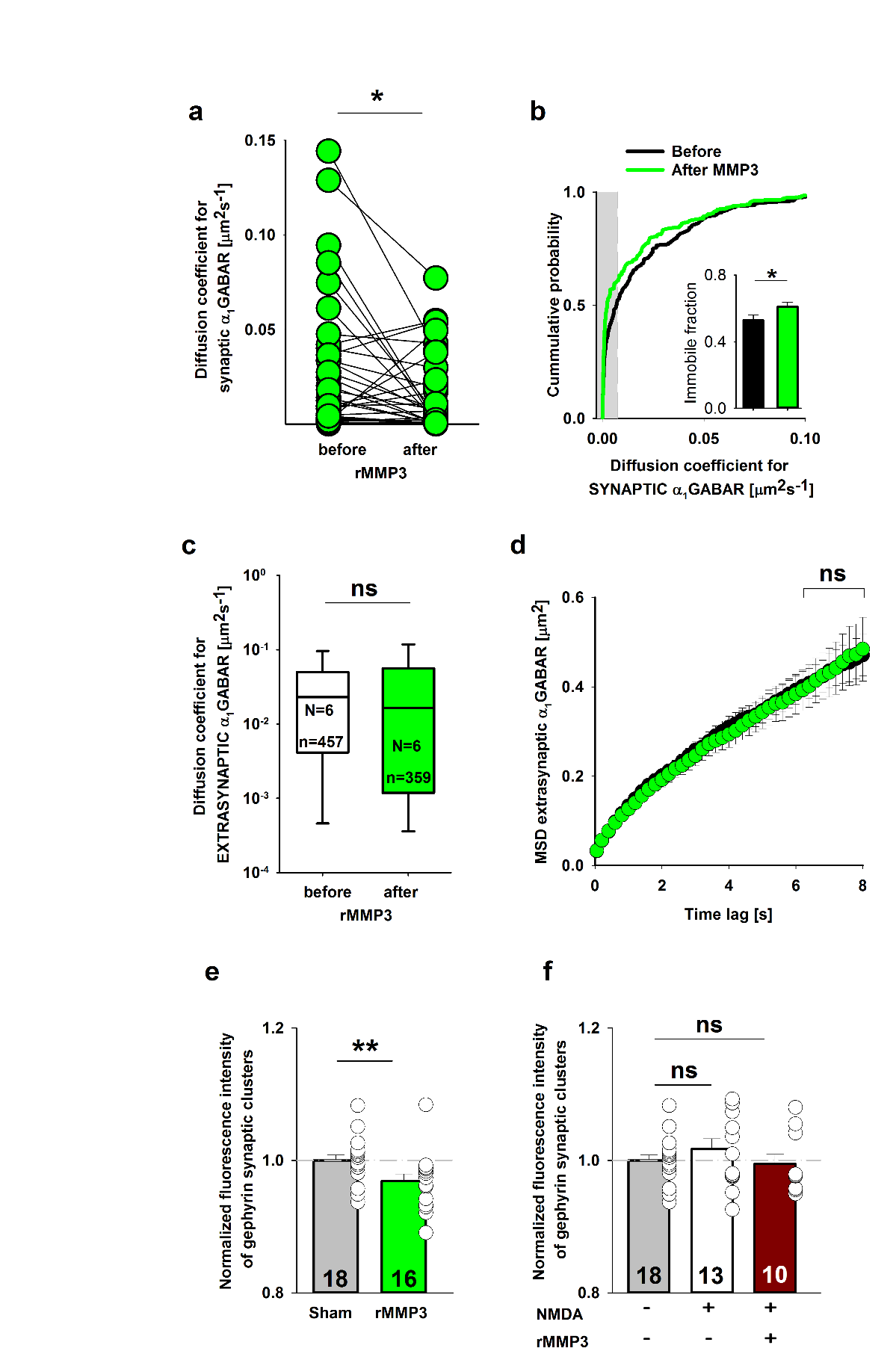


**Supplementary Fig. 7. The impact of application of exogenous active rMMP3 on diffusion parameters of synaptic, extrasynaptic α_1_GABA_A_ receptors and mean fluorescence intensity of gephyrin clusters**

1. Coefficients of diffusion at synapses of the same GABA_A_ receptors identified before and after rMMP3 treatment (n = 45 pairs of QDs, p = 0.015, Wilcoxon signed-rank test).
2. Cumulative probability distributions of diffusion coefficients for synaptic α_1_GABA_A_ receptors before (black) and 20 min after (green) the application of rMMP3. The gray area marks the part of the cumulative distribution that contains immobile receptors (D < 0.0075 µm^2^ s^-1^). (Inset) Average immobile fraction before and after MMP3 treatment.
3. Interquartile range (IQR; 25-75%) and median diffusion coefficient of synaptic α_1_GABA_A_ receptors before and 20 min after short-term (2 min) incubation with active MMP3. N and n refer to the number of coverslips and analyzed trajectories, respectively. N and n refer to the number of coverslips and analyzed trajectories, respectively
4. Lack of significant difference in mean square displacement of extrasynaptic α_1_GABA_A_ receptors before (black) and 20 min after rMMP3 incubation (green).
5. The mean fluorescence intensity of gephyrin clusters at dendritic inhibitory synapses (normalized to sham).
6. Quantification of the mean fluorescence intensity of synaptic gephyrin clusters in neuronal cultures that were treated with sham solution, NMDA, and NMDA + rMMP3.

*p < 0.05, **p < 0.01, ns - nonsigniﬁcant. The data in b-f were analyzed using the Mann-Whitney U-test with comparisons vs. the respective controls. The numbers in the bars refer to the number of recordings.


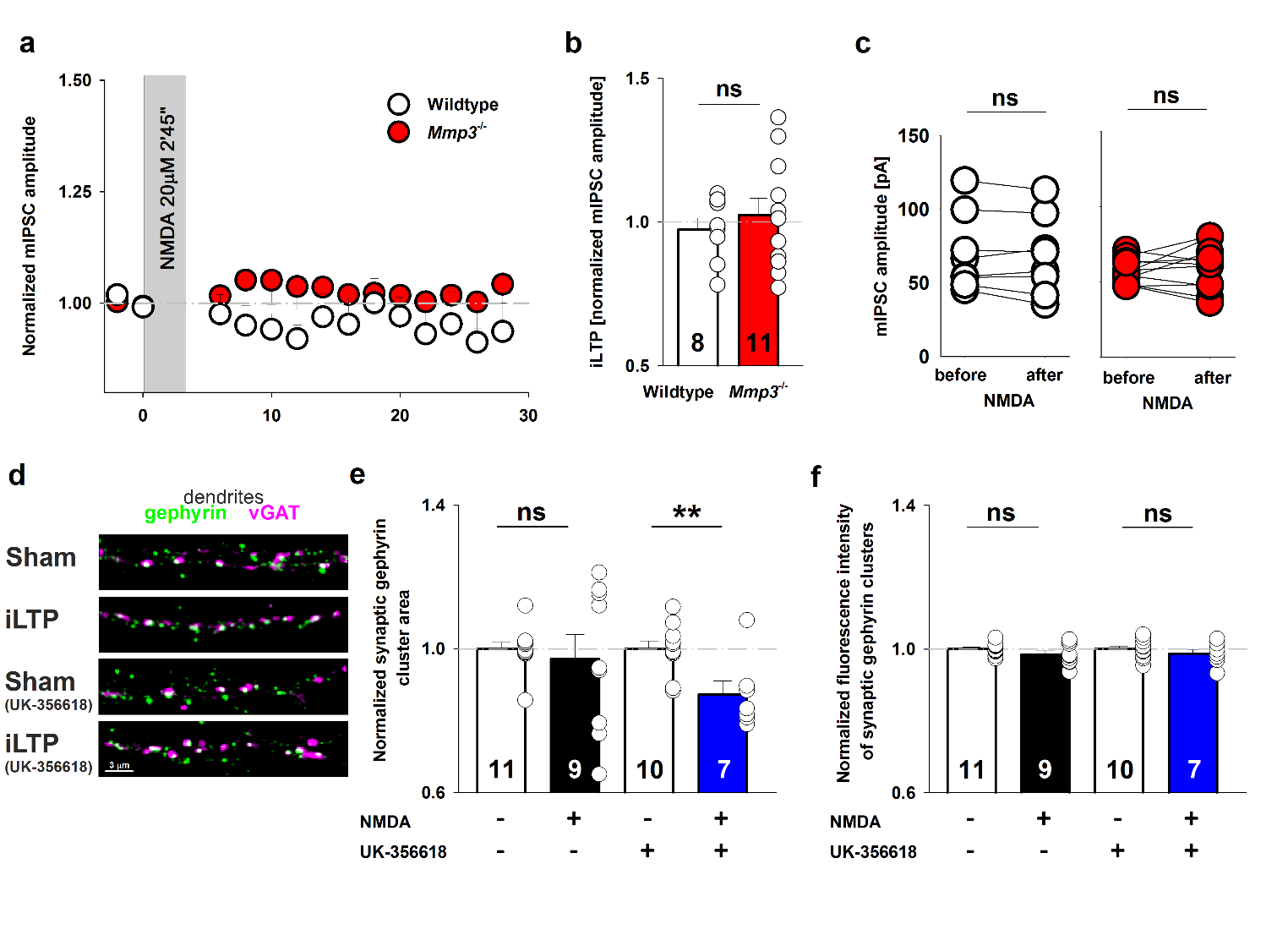


**Supplementary Fig. 8. Extended NMDA stimulation in neuronal cultures does not rescue impaired iLTP in *Mmp3*^-/-^ neurons.**

**(a-c)** mIPSC amplitude (**a**) recorded in hippocampal neuronal cultures prepared from wildtype (white) and *Mmp3*^-/-^ mice (red). Gray area marks the application of NMDA that lasts for 2 min 45 s. Note that to induce stable iLTP NMDA should be applied in our experiments for 2 min 15 s. Longer NMDA superfusion (**b,c**) is not efficient in iLTP induction in wildtype neurons and does not rescue impaired iLTP in MMP3 deficient.

**(d)** Representative confocal maximum projection images obtained from wildtype neuronal cultures immunolabeled with antibodies to presynaptic vGAT and postsynaptic gephyrin. The colocalization of gephyrin and vGAT (white) corresponds to the presence of gephyrin at GABAergic synapses.

**(e and f)** Quantification of the synaptic gephyrin clusters area (**e**) and normalized total fluorescence intensity (**f**) in neuronal cultures treated with respective sham solution, NMDA for 2 min 45 s (black) and NMDA for 2 min 45 s with UK356618 (blue). Note, unchanged area of gephyrin synaptic clusters when NMDA is applied for 2 min 45 s instead of 2 min 15 s. Additionally, superfusion of NMDA for 2 min 45 s in the presence of UK356618 leads to significant decrease in gephyrin synaptic cluster area.

**P < 0.01; ns - nonsigniﬁcant. *t*-test vs. respective control (**b, e, f**); paired *t* test (**c**); Numbers on bars refer to the number of used coverslips from at least three different batch of cultures.
